# Effect of Digital Technology–Based Interventions on Core Symptoms and Cognitive Function in Children With Attention-Deficit/Hyperactivity Disorder: Systematic Review and Meta-Analysis

**DOI:** 10.2196/103353

**Published:** 2026-10-09

**Authors:** Aiwei Wang, Weijie Qin, Lu Chen, Wei Tuo

**Affiliations:** 1 College of Physical Education, Yangzhou University, No. 196 Huayang West Road, Hanjiang District, Yangzhou, Jiangsu, 225127, China, 86 13952569475

**Keywords:** attention-deficit/hyperactivity disorder, ADHD, children, digital technology–based interventions, core symptoms, cognitive function, meta-analysis

## Abstract

**Background:**

Attention-deficit/hyperactivity disorder (ADHD) is a common neurodevelopmental disorder in children, characterized by core symptoms of hyperactivity, impulsivity, and inattention, in addition to cognitive impairments that compromise physical and psychological development. Digital technology–based interventions have emerged as a promising approach for ameliorating both core symptoms and cognitive impairments. However, a comprehensive evidence base supporting their efficacy is lacking.

**Objective:**

This study aimed to systematically evaluate the effects of digital interventions on core symptoms and cognitive impairments in children with ADHD.

**Methods:**

Web of Science, PubMed, EBSCOhost, and ProQuest were systematically searched using predefined inclusion and exclusion criteria. The risk of bias of the included studies was assessed using the revised Cochrane risk-of-bias tool for randomized trials. Effect sizes were pooled under a random-effects model, and heterogeneity across studies was evaluated using the *I*² statistic. Publication bias was assessed using Egger regression and Begg rank correlation tests. Subgroup analyses were performed to explore sources of heterogeneity across intervention type, age, intervention dosage, control group type, and outcome measurement tools. Sensitivity analyses were performed by switching from a random-effects model to a fixed-effects model to confirm the results’ robustness.

**Results:**

Thirty-seven randomized controlled trials were included, encompassing 4 types of digital interventions: computer-based interventions, serious video games, exergames, and virtual reality. Digital interventions significantly alleviated core symptoms and cognitive impairments, with improvements in the former primarily attributable to serious video games (*P*=.02) and those in the latter mainly attributable to computer-based interventions (*P*=.04) and serious video games (*P*=.02). Subgroup analyses revealed that effects were stronger in younger children (<10 years) and with short-to-moderate intervention duration (≤8 weeks) and were influenced by control group type and assessment modality.

**Conclusions:**

Digital interventions may relieve core symptoms and cognitive deficits in children with ADHD, with highly variable efficacy shaped by intervention type, age, dosage, and assessment methods. Serious games produce superior improvements in core symptoms, whereas computer-based training better enhances cognitive function. Future trials should optimize design and implement stratified analyses (eg, by symptom subtype and age) to clarify tailored intervention effects for better clinical application.

## Introduction

Attention-deficit/hyperactivity disorder (ADHD) is a common neurodevelopmental disorder in children, affecting approximately 5.2% of children globally [1] and 6% of children in China [2]. It is characterized by inattention, hyperactivity, and impulsivity as its core symptoms [3]. Inattention manifests as difficulty sustaining attention and increased distractibility [4]. For example, during a 10-minute independent seatwork period, children with ADHD only spend about one third as much time on-task as typically developing children [5]. Hyperactivity-impulsivity manifests as fidgeting, excessive motor activity, impulsive behaviors, and poor behavioral control [6]. Compared with typically developing children, children with ADHD exhibit 6 times higher hyperactivity-impulsivity scores and significant impairments in social, academic, and daily functioning [7].

Compared with typically developing children, children with ADHD also exhibit impaired cognitive function [8], performing 1.0 to 1.5 SDs lower on working memory tasks, corresponding to 20% to 30% lower accuracy [9]; significantly impaired cognitive flexibility, as evidenced by slower switching and greater switch costs when task rules change [10]; and significantly lower information processing speed (mean 48.91, SD 27.05 vs mean 75, SD 15 in typically developing children) [11]. Consequently, at school, they face greater difficulty remembering task instructions, executing complex planning tasks, and completing homework on time, which impairs academic performance.

ADHD-associated core symptoms and cognitive impairments compromise not only academic performance but also social competence and long-term development [12], highlighting the need for early intervention. Currently, combined treatment approaches integrating pharmacotherapy with psychological interventions represent the mainstream clinical paradigm for ADHD [13]. However, both pharmacological and behavioral interventions have notable limitations. Evidence indicates that behavioral interventions, although effective, are time-consuming, costly, and not easily accessible to all children who need them [11,14]. Pharmacotherapy, meanwhile, is highly effective for core symptoms but has limited impact on cognitive function, underscoring the need for alternative treatments. Thus, nonpharmacological therapies may be viable alternatives to alleviate both core symptoms and cognitive impairments in children with ADHD [15].

With advances in technology, digital technology–based interventions [16], such as computer-based interventions, virtual reality (VR), serious video games, and exergames, have emerged and shown potential to ameliorate core symptoms and cognitive impairments in children with ADHD [17-19]. However, as research in this area is nascent, findings remain inconclusive. Studies testing computer-based interventions to improve attention-related core symptoms have reported inconsistent results [17,20,21]. Similar inconsistencies have been observed for serious video games: a study found that serious game-based working-memory training significantly ameliorated inattention in children with ADHD [22], whereas another reported no significant improvement in core symptom-related outcomes [23]. Digital interventions have also shown potential to improve cognitive outcomes [24,25] and promote sustained enhancement of cognitive and daily adaptive functions in children with ADHD [26]. Nevertheless, some studies have demonstrated that computer-based interventions do not significantly improve overall cognitive function [27].

Given the diverse digital technology–based interventions and the inconsistent findings across studies, no definite conclusion has been reached. Systematic review-based integration and synthesis of the intervention effects are therefore warranted to clarify the interventions’ true effects on children with ADHD and provide an evidence base for future studies [28]. To date, 5 review articles have examined the effects of digital interventions on core symptoms and cognitive function in children with ADHD [15,29-32]. One was a comparative review, including 36 studies, which suggested that video games have the potential to improve core symptoms in children with ADHD aged 8 to 12 years [29]. The other 3 were systematic reviews evaluating the effects of serious games or video games on core symptoms and cognitive functions in children (≤18 years) with ADHD; they reported that these approaches can alleviate core symptoms and cognitive impairments [30-32]. On this basis, Wong et al [15] provided meta-analytic evidence that technology-based interventions improve attention and executive function, but not hyperactivity/impulsivity, in children with ADHD aged 6 to 12 years, upgrading the level of evidence from narrative reviews to pooled effect sizes.

The findings of existing reviews further substantiate the potential of digital technology–based interventions to improve core symptoms and cognitive function in children with ADHD. However, all 5 reviews focused on only 1 or 2 types of digital technology modalities and did not provide a comprehensive comparison across all major digital intervention types [15,29-32]. Moreover, due to the heterogeneity of the included studies, most of the reviews were limited to descriptive analyses without quantitative synthesis [32], and the only meta-analysis included was restricted to children aged 6 to 12 years [15], leaving the effects on adolescents (13‐18 years) unexplored.

To address these gaps, we conducted a systematic review and meta-analysis with the aims to (1) comprehensively evaluate the effects of digital technology–based interventions on core symptoms and cognitive function in children aged 5 to 18 years with ADHD, (2) examine the differences in the effects of various digital intervention modalities, and (3) explore potential moderators (eg, age, intervention types, dosage, control type, and measurement tools) through subgroup analyses. Additionally, as randomized controlled trials (RCTs) are considered the gold standard for examining intervention effectiveness, we only included RCTs in this study [33].

To our knowledge, this is the first systematic review and meta-analysis to simultaneously examine all types of digital technology–based interventions (ie, computer-based interventions, serious games, video games, exergames, and VR) and directly compare their effects on core symptoms and cognitive function in both young children and adolescent children with ADHD. This comprehensive approach enables clinicians and researchers to make evidence-based decisions regarding the selection of specific digital intervention modalities for different therapeutic targets.

## Methods

### Study Design and Registration

This study was designed, conducted, and reported in accordance with the PRISMA (Preferred Reporting Items for Systematic Reviews and Meta-Analyses) statement [34]. Checklist 1 provides the complete PRISMA checklist. This review was prospectively registered with PROSPERO (International Prospective Register of Systematic Reviews, CRD420261399517).

### Search Strategy

We systematically searched Web of Science, PubMed, EBSCOhost, and ProQuest for relevant literature from inception through January 2026 [35]. This systematic review and meta-analysis was designed following the PICOS (population, intervention, comparison, outcome, study design) framework. Keywords were organized into 3 categories: population (eg, children, adolescents, ADHD, or attention-deficit/hyperactivity disorder), intervention (eg, digital technology–based intervention, serious video game, exergame, or virtual reality), and outcomes (eg, core symptoms or cognitive function). Multimedia Appendix 1 presents the complete search strategy.

### Literature Screening

Initially retrieved records were imported into EndNote (Clarivate Analytics) for deduplication. Thereafter, titles and abstracts were screened to identify potentially eligible studies. Full texts of the remaining records were then retrieved and assessed for final inclusion. The inclusion criteria were as follows: (1) participants were children with ADHD, (2) they were 5 to 18 years old [36], (3) the intervention was based on a digital technology (eg, serious video games, computer-based interventions, or VR), (4) at least one core symptom-related or cognitive function-related outcome was reported, (5) the article was published in English in a peer-reviewed journal with full text available, and (6) the study was an RCT. The exclusion criteria were (1) review articles and (2) articles with no reported results. Two researchers independently performed the literature search and screening. Any disagreements were resolved through discussion with the corresponding author to reach consensus. Reasons for full-text exclusion were documented and are provided in Multimedia Appendix 1, in accordance with PRISMA requirements [28].

### Risk of Bias Assessment

The risk of bias in the included studies was assessed using the revised Cochrane risk-of-bias tool for randomized trials (RoB 2.0), which evaluates 5 domains: the randomization process, deviations from intended interventions, missing outcome data, outcome measurement, and selection of the reported result [37]. Each domain was rated as “low risk of bias,” “some concerns,” or “high risk of bias.” A study was judged to have an overall “high risk of bias” if any domain was rated as high risk. If no domain was rated as high risk but at least one raised “some concerns,” the overall rating was “some concerns” or “high risk of bias,” depending on the pattern of concerns. Two researchers independently performed the assessment, and any disagreements were resolved by the corresponding author, who made the final decision.

### Data Extraction and Data Analysis

The following data were extracted from the included studies: author information, study design, population characteristics, intervention type, intervention schedule, and primary outcome measures.

A meta-analysis was performed using Review Manager (RevMan; version 5.4; The Cochrane Collaboration). Effect sizes were pooled under a random-effects model [38]. Heterogeneity across studies was evaluated using the *I*² statistic, with values of ≤25%, 25% to 50%, and ≥75% indicating low, moderate, and high heterogeneity, respectively [39]. To systematically explore potential sources of heterogeneity, subgroup analyses were conducted based on the following exploratory variables (when data were available): (1) intervention type: computer-based interventions, serious video game, exergame, and VR technology; (2) age group: early school-age (<10 years), late school-age/preadolescent (10‐11.9 years), and adolescent (≥12 years); (3) intervention dosage: total duration (short: ≤4 weeks, medium: 5‐8 weeks, long: >8 weeks), training frequency (low: 1‐3 sessions/week, high: ≥4 sessions/week), and session time (short: ≤20 minutes, medium: 21‐45 minutes, long: >45 minutes); (4) control group type: treatment-as-usual, active control, waitlist/blank control; and (5) outcome measurement: subjective report (parent/teacher ratings) vs objective tests. Publication bias was assessed using Egger regression and Begg rank correlation tests in Stata 18.0 [40]. Sensitivity analyses were conducted by switching from the random-effects model to a fixed-effects model to examine the robustness of the meta-analysis results [41].

## Results

### Literature Search

Overall, 1330 articles were initially retrieved from the 4 databases. After removing 248 duplicates, the titles and abstracts of the remaining records were screened, with 913 being excluded for irrelevance. The full texts of the remaining 169 articles were then assessed against the inclusion and exclusion criteria. Ultimately, 37 studies met the eligibility criteria and were included. Figure 1 presents the flow diagram of the literature search and screening process.

**Figure 1.**
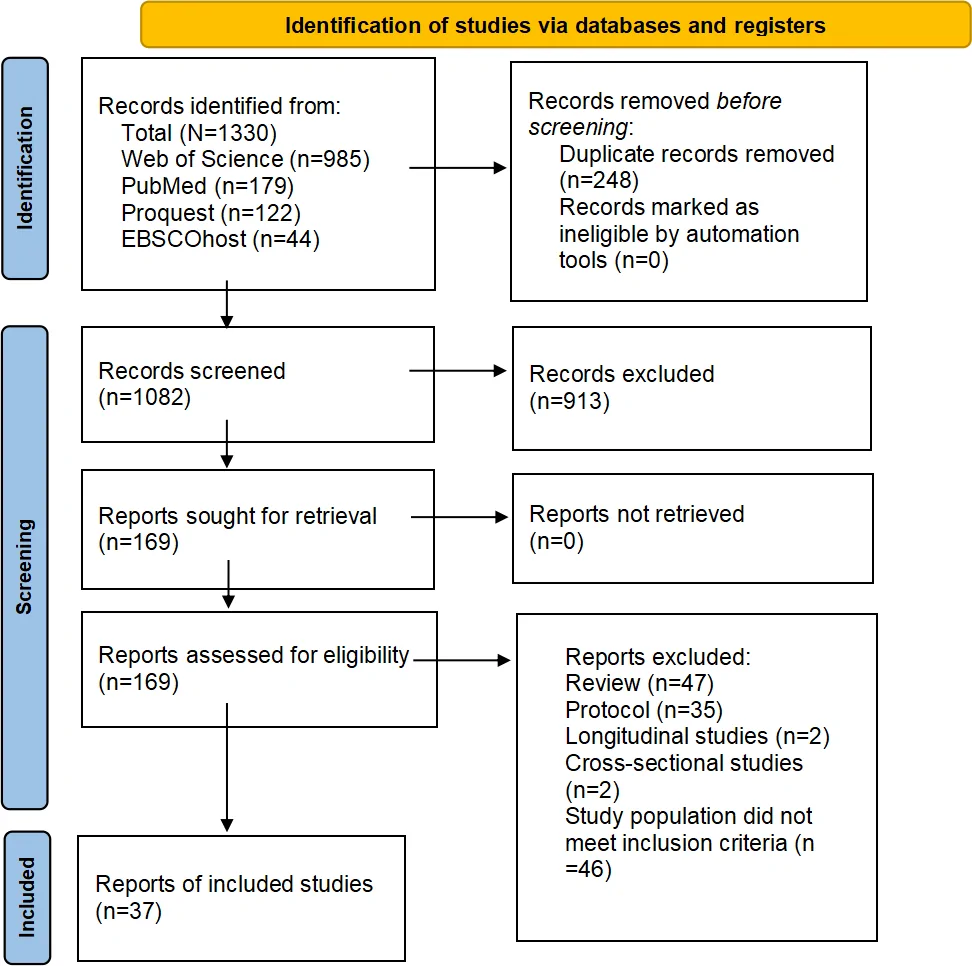
Flowchart of literature search and screening.

### Study Characteristics

The meta-analysis included 37 studies published from 2002 through 2024, encompassing 2149 children with ADHD aged 5 to 18 years. Among these, 26 studies used a 2-arm trial design, while the remaining 11 used a 3-arm design [10,21,42-50].

The included studies were conducted across 12 countries: 8 in the United States [21,23,51-56]; 5 in China [35,46,48,50,57]; 2 in Denmark [14,17]; 2 in Sweden [58,59]; 2 in Switzerland [60,61]; 5 in the Netherlands [10,11,49,62,63]; 3 in Iran [22,42,64]; 4 in Spain [47,65-67]; 3 in South Korea [44,45,68]; and 1 each in Belgium [69], France [43], and Israel [18].

The included studies were conducted in various settings: 14 in schools, 10 in communities [10,11,18,22,43,57,62,66,67,69], 9 in homes [14,17,51-54,58,60,61], 3 in laboratories [44,49,65], and 1 in a hospital [35].

The interventions were based on computers (16 studies [14,21,23,42,46,49,51-56,58,59,65,67]), serious video games (9 studies [10,11,17,18,22,62,64,66,69]), exergames (3 studies [57,60,61]), and VR (9 studies [35,43-45,47,48,50,63,68]). Control conditions included waitlist control (12 studies [18,21,22,35,44,45,50,51,61,64,66,69]), treatment as usual (4 studies [17,23,56,65]), placebo control (5 studies [10,14,42,52,59]), blank control (5 studies [22,48,49,55,68]), physical activity (1 study [57]), and other digital technology controls (10 studies [11,43,47,53,54,58,60,62,63,67]). The intervention designs were long-term (≥12 weeks) in 13 studies [11,18,21-23,46,48,51,56,58,64,66,67] and short-term (<12 weeks) in 24 studies. Nine studies [11,18,22,42,46,48,49,58,65] administered long-duration sessions (≥1 hour), and 28 [10,14,17,21,23,35,43-45,47,50-57,59-64,66-69] administered short-duration sessions (<1 hour). Fourteen studies [10,11,17,18,22,42,46,47,51-53,58,65,69] reported follow-up assessments, with a follow-up period of 12 weeks or above in 10 studies [10,17,18,22,42,46,47,51,53,58] and less than 12 weeks in 4 studies [11,52,65,69]. Multimedia Appendix 2 presents detailed study characteristics.

### Risk of Bias Assessment

Figure 2 presents the risk of bias assessment for the included RCTs. Of the 37 studies, 11 had a low risk of bias and 26 had some concerns; none had a high risk. Of the 5 domains, the randomization process showed the lowest risk of bias. Some concerns were identified for missing outcome data in 10.8% (n=4) of the studies, for deviations from intended interventions in 32.4% (n=12), and for selection of the reported result in 32.4% (n=12). The highest proportion of “some concerns” was observed in the outcome measurement domain (n=15, 41%). Overall, approximately 30% (n=11) of the studies had a low risk, and 70% (n=26) had some concerns, indicating a generally acceptable overall risk of bias.

**Figure 2.**
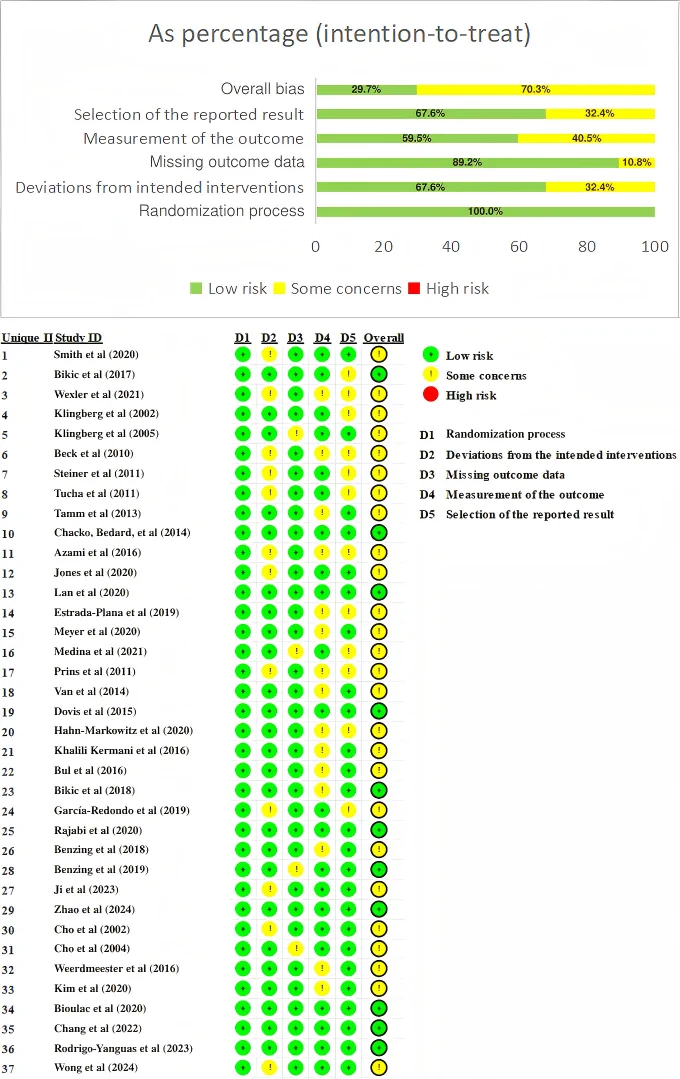
Methodological quality assessment of the included studies [10,11,14,17,18,21-23,27,35,42-51,53-69].

### Outcomes and Effects

#### Core Symptoms

Overall, 28 studies—14 using computer-based interventions, 7 using serious video game interventions, and 7 using VR interventions—assessed core symptoms. Figure 3 presents the meta-analysis results for core symptoms.

**Figure 3.**
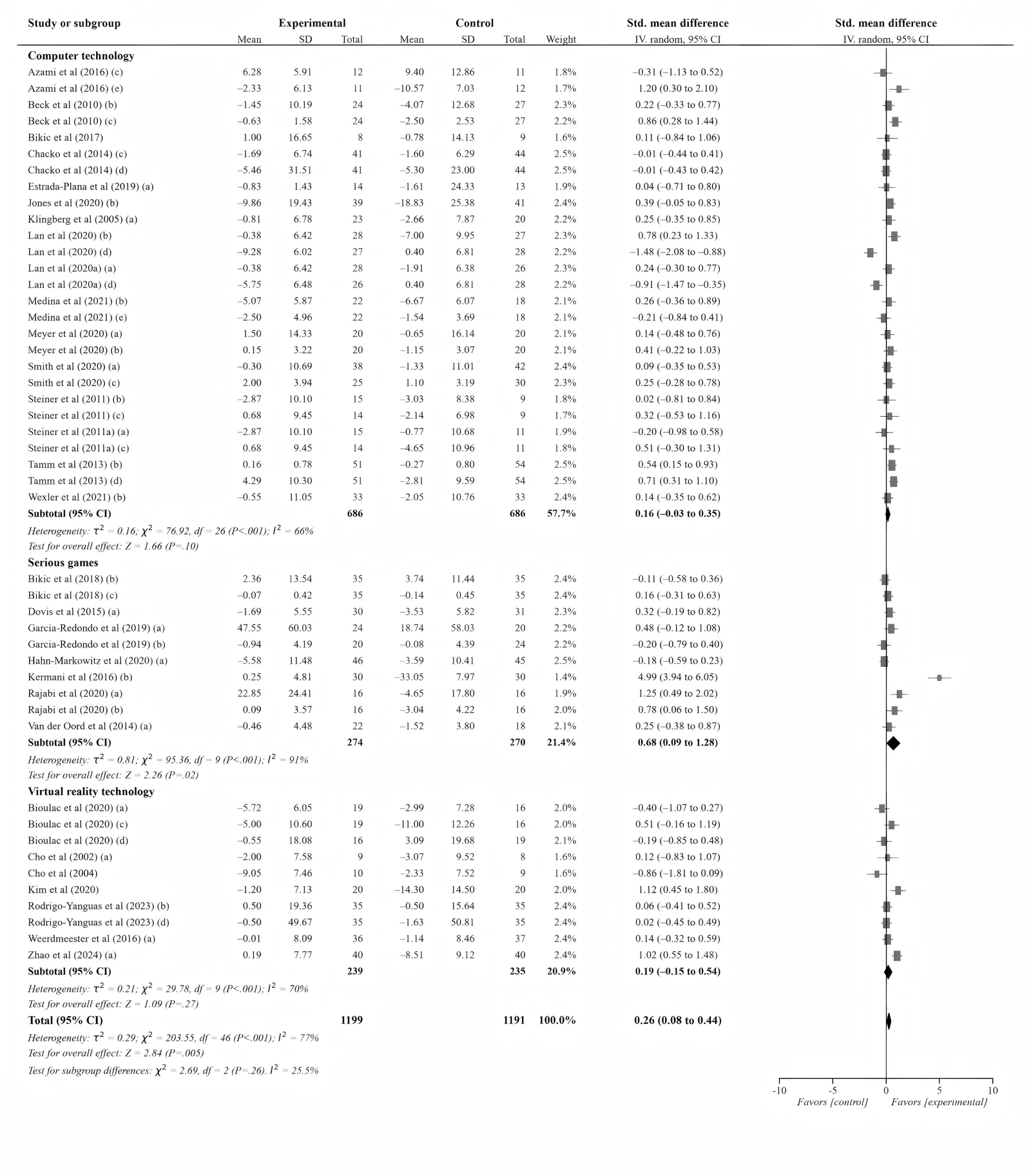
Meta-analysis results for core symptoms. Lowercase letters in parentheses appended after the study label (eg, (a), (b), (c), (d)) denote different outcome measurement instruments/rating scales used within that study [10,14,17,18,21-23,35,42-47,51-56,58,63-69]. Std: standard.

The overall effect of digital technology–based interventions on core ADHD symptoms was statistically significant (standardized mean difference [SMD]=0.26, 95% CI 0.08-0.44; *I*²=77%; *P*=.005). Subgroup analyses by intervention type indicated that neither computer-based interventions (SMD=0.16, 95% CI −0.03 to 0.35; *I*²=66%; *P*=.10) nor VR interventions (SMD=0.19, 95% CI −0.15 to 0.54; *I*²=70%; *P*=.27) yielded statistically significant improvements in core symptoms. By contrast, serious video game interventions produced a significant improvement (SMD=0.68, 95% CI 0.09-1.28; *I*²=91%; *P*=.02).

#### Subgroup Analyses for Core Symptoms

Given the substantial overall heterogeneity (*I*²=77%), predefined subgroup analyses were conducted to identify potential heterogeneity sources, with stratification by age, intervention dosage, control design, and outcome measurement approach (Table 1).

**Table 1.** Subgroup analysis results for core symptoms^a^.

Type and subgroup	Number of effect sizes	SMD^b^ (95% CI)	*I*^2^^c^ (%)	*P* value
Intervention type				
Computer technology	27	0.16 (−0.03 to 0.35)	66	.10
Serious games	10	0.68 (0.09 to 1.28)	91	.02
Virtual reality technology	10	0.19 (−0.15 to 0.54)	70	.27
Mean age (years)				
Teenagers (≥12)	9	0.03 (−0.20 to 0.26)	0	.79
Late school age/preadolescence (10‐11.9)	12	0.15 (−0.27 to 0.57)	85	.49
Early school-age children (<10)	24	0.36 (0.10 to 0.62)	82	.007
Period (weeks)				
Short (≤4)	8	0.27 (−0.15 to 0.69)	67	.2
Long (>8)	21	0.28 (−0.08 to 0.63)	86	.13
Medium (5‐8)	19	0.26 (0.10 to 0.43)	40	.002
Frequency				
Low (1‐3 sessions/week)	26	0.44 (0.16 to 0.71)	81	.002
High (≥4 sessions/week)	18	0.11 (−0.03 to 0.25)	12	.11
Session time (minutes)				
≤20	10	−0.07 (−0.53 to 0.38)	81	.75
>45	14	0.28 (−0.21 to 0.77)	90	.26
21‐45	21	0.4 (0.22 to 0.57)	49	.001
Control group				
Regular training	9	0.17 (−0.03 to 0.36)	7	.09
Active control	13	−0.1 (−0.39 to 0.19)	74	.49
Blank control	23	0.45 (0.14 to 0.75)	82	.004
Outcome measurement				
Subjective	33	0.29 (0.09 to 0.50)	77	.005
Objective	15	−0.03 (−0.37 to 0.31)	78	.87

a Positive standardized mean difference favors intervention; significant if 95% CI excludes 0 and *P*<.05.

b SMD: standardized mean difference.

c *I²*: heterogeneity.

Age subgroup analyses showed significant intervention benefits exclusively in children aged less than 10 years (SMD=0.36, 95% CI 0.10‐0.62; *I*²=82%; *P*=.007). No significant effects were found in late school-age/preadolescent children, and adolescents aged 12 years or above exhibited no measurable improvements.

For intervention dosage metrics, only 5- to 8-week medium-term interventions produced significant effects with the lowest heterogeneity (SMD=0.26; *I*²=40%; *P*=.002), while shorter (≤4 weeks) and longer (>8 weeks) interventions showed no statistical significance. A counterintuitive frequency-related result was observed: low-frequency training (1‐3 sessions/week) yielded significant benefits (SMD=0.44; *I*²=81%; *P*=.002), whereas high-frequency training showed no significant effects but markedly lower heterogeneity. This discrepancy is likely confounded by cross-study variations in intervention types [62]. Regarding single-session duration, 21- to 45-minute sessions generated the largest and most robust effects (SMD=0.40; *I*²=49%; *P*<.001), whereas shorter and longer sessions were ineffective, with extremely high heterogeneity for long-duration sessions. Overall, interventions with 21‐ to 45-minute sessions and a 5‐ to 8-week course represent the most stable and evidence-based dosage regimen, while frequency-associated findings require further rigorous validation.

Control subgroup analyses indicated that waitlist/blank controls yielded the largest pooled effect size (SMD=0.45; *I*²=82%; *P*=.004), while treatment-as-usual controls showed a marginally significant trend. Active controls demonstrated no intervention benefits and no beneficial trend. Effect sizes declined progressively with stricter control designs, confirming that control selection substantially affects the magnitude of pooled intervention effects in meta-analyses.

Subgroup stratification by measurement type revealed significant improvements in parent- and teacher-reported subjective outcomes (SMD=0.29; *I*²=77%; *P*=.005), whereas objective behavioral and cognitive tests showed no significant changes (SMD=−0.03; *I*²=78%; *P*=.87). This subjective-objective discrepancy implies that reported symptomatic improvements may partly stem from rater expectancy bias, rather than reflecting genuine objective behavioral changes induced by interventions.

#### Cognitive Function

Thirty-one studies—14 using computer-based interventions, 8 using serious video game interventions, 3 using exergame interventions, and 6 using VR interventions—assessed cognitive function. Figure 4 presents the meta-analysis results.

**Figure 4.**
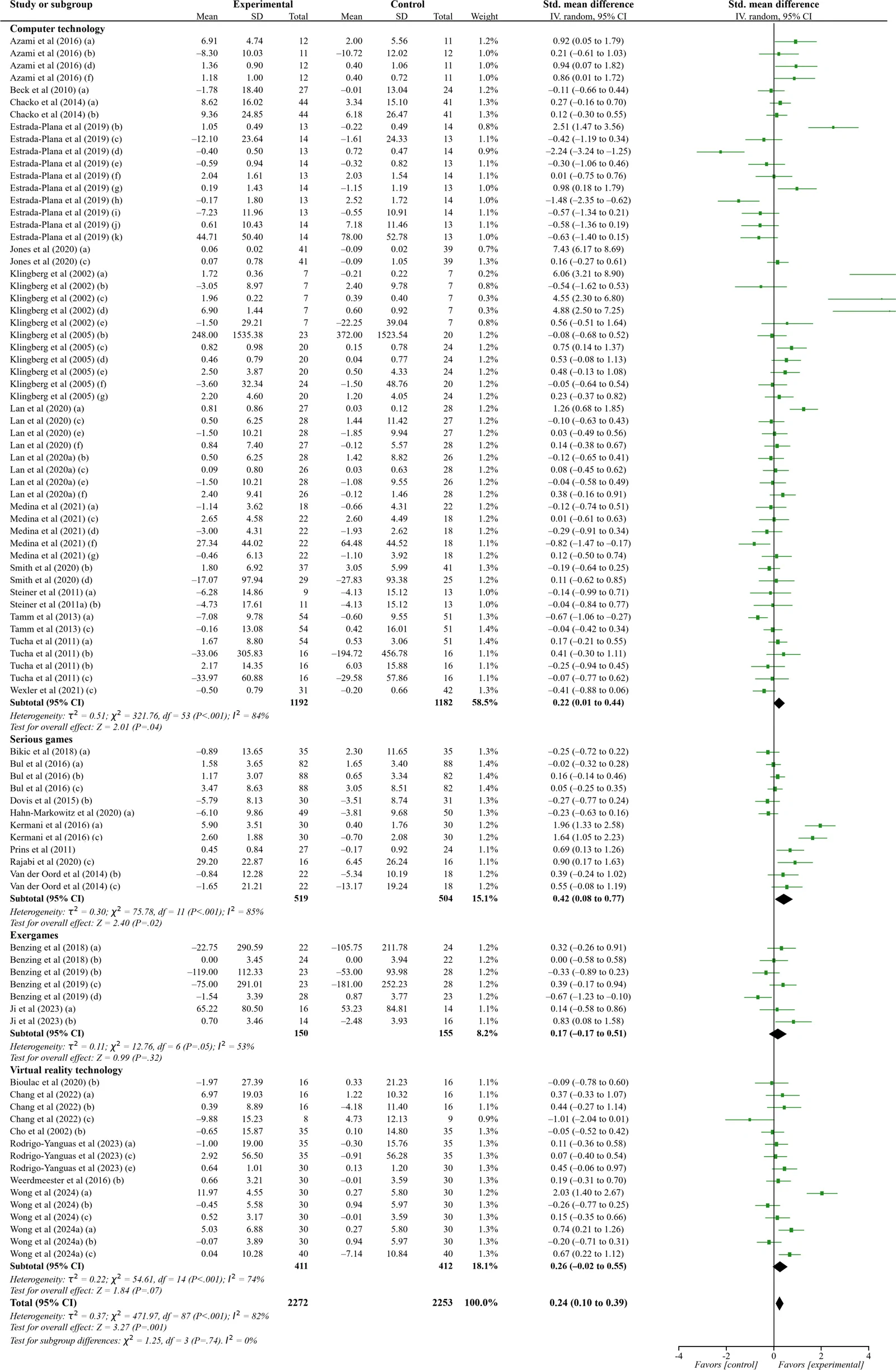
Meta-Analysis results for cognitive function. Lowercase letters in parentheses appended after the study label (eg, (a), (b), (c), (d)) denote different outcome measurement instruments/rating scales used within that study [10,11,17,18,21-23,42,43,45-53,55-65,67,69]. Std: standard.

Overall, digital technology–based interventions significantly improved cognitive function in children with ADHD (SMD=0.24, 95% CI 0.10-0.39, *I*²=82%; *P*=.001). Subgroup analyses by intervention type revealed that these improvements were afforded by computer-based (SMD=0.22, 95% CI 0.01-0.44, *I*²=84%; *P*=.04) and serious video game interventions (SMD=0.42, 95% CI 0.08-0.77, *I*²=85%; *P*=.02), not by VR (SMD=0.26, 95% CI −0.02 to 0.57, *I*²=74%; *P*=.07) or exergame interventions (SMD=0.17, 95% CI −0.17 to 0.51, *I*²=53%; *P*=.32).

#### Subgroup Analyses for Cognitive Function

Subgroup analyses of core symptoms were conducted stratified by age, dosage parameters, control-group type, and outcome measurement tools, with results shown in Table 2.

**Table 2.** Subgroup analysis results for cognitive function^a^.

Type and subgroup	Number of effect sizes	SMD^b^ (95% CI)	*I*²^c^ (%)	*P* value
Intervention type				
Computer technology	52	0.22 (0.01 to 0.44)	84	.04
Serious games	12	0.42 (0.08 to 0.77)	85	.02
Exergames	7	0.17 (−0.17 to 0.51)	53	.32
Virtual reality technology	16	0.26 (−0.02 to 0.55)	74	.07
Mean age (years)				
Teenagers (≥12)	6	−0.03 (−0.27 to 0.20)	0	.78
Late school age/preadolescence (10‐11.9)	18	0.31 (−0.02 to 0.64)	75	.07
Early school-age children (<10)	54	0.19 (0.02 to 0.36)	80	.03
Period (weeks)				
Short (≤4)	21	0.33 (0.08 to 0.59)	71	.01
Long (>8)	31	0.15 (−0.03 to 0.33)	72	.09
Medium (5‐8)	37	0.14 (−0.09 to 0.37)	79	.22
Frequency				
Low (1‐3 sessions/week)	58	0.13 (−0.02 to 0.28)	74	.09
High (≥4 sessions/week)	28	0.28 (0.03 to 0.53)	78	.03
Session time (minutes)				
≤20	17	0.08 (−0.19 to 0.35)	75	.57
>45	42	0.16 (−0.03 to 0.34)	77	.10
21‐45	30	0.30 (0.08 to 0.52)	75	.008
Control group				
Regular training	19	0.47 (0.14 to 0.80)	75	.005
Active control	34	0.19 (0.02 to 0.35)	66	.02
Blank control	36	0.07 (−0.14 to 0.28)	81	.53
Outcome measurement				
Subjective	34	0.12 (−0.06 to 0.30)	75	.19
Objective	55	0.23 (0.06 to 0.40)	76	.009

a Positive standardized mean difference favors intervention; significant if 95% CI excludes 0 and *P*<.05.

b SMD: standardized mean difference.

c *I²*: heterogeneity.

Age-stratified subgroup analyses for cognitive outcomes revealed significant intervention gains in early school-age children aged less than 10 years (SMD=0.19, 95% CI 0.02-0.36; *I*^2^=80%; *P*=.03). Late school-age/preadolescent children (10‐11.9 years) showed a marginally significant positive trend (SMD=0.31, 95% CI −0.02 to 0.64; *I*^2^=75%; *P*=.07), while no apparent cognitive improvements were observed in adolescents aged 12 years or above. The young child subgroup exhibited high heterogeneity with variable effect sizes, whereas adolescent studies yielded consistent null effects.

Dosage parameter analyses indicated that only short-term interventions (≤4 weeks) produced significant and maximal cognitive benefits (SMD=0.33, 95% CI 0.08-0.59; *I*^2^=71%; *P*=.01). Neither medium-term (5‐8 weeks) nor long-term (>8 weeks) training achieved statistical significance, suggesting that cognitive improvements may depend on early intervention activation rather than prolonged training accumulation. High-frequency intervention (≥4 sessions/week) generated significant effects (SMD=0.28, 95% CI 0.03-0.53; *I*^2^=78%; *P*=.03), while low-frequency training (1‐3 sessions/week) was nonsignificant, supporting an expected dose-response relationship. Sessions of 21‐45 minutes yielded the optimal and significant cognitive effects (SMD=0.30, 95% CI 0.08-0.52; *I*^2^=75%; *P*=.008), whereas shorter (≤20 minutes) and longer (>45 minutes) sessions showed no efficacy. Collectively, intervention regimens characterized by short duration, high frequency, and moderate single-session length are optimal for improving cognitive function.

Gradient effect sizes were observed across control conditions. The treatment-as-usual control group showed the largest effects (SMD=0.47, 95% CI 0.14-0.80; *I*^2^=75%; *P*=.005), followed by active controls (SMD=0.19, 95% CI 0.02-0.35; *I*^2^=66%; *P*=.02). Waitlist/blank controls yielded the smallest and nonsignificant effects, demonstrating that the rigor of control group design directly modulates pooled effect size estimates.

For outcome measurement modalities, objective tests detected significant intervention-induced cognitive improvements (SMD=0.23, 95% CI 0.06-0.40; *I*^2^=76%; *P*=.009), while subjective ratings showed no statistical significance. This finding contrasts distinctly with core symptom outcomes, indicating that objective assessments are more sensitive to training-related cognitive changes, whereas caregiver subjective evaluations are less responsive to cognitive than behavioral alterations. High heterogeneity persisted in both objective and subjective subgroups, suggesting substantial between-study variability across measurement approaches [70].

#### Publication Bias Assessment

Publication bias for core symptom-related and cognitive function–related outcomes was assessed using Egger regression and Begg rank correlation tests, with Hedges *g* as the effect size. The Egger test was significant for core symptoms (*P*=.02) and cognitive function (*P*=.01), but the Begg test was not (*P*=.18 and *P*=.20, respectively). This discrepancy suggests some degree of publication bias in the studies that reported these outcomes. However, given the high between-study heterogeneity (*I*²>75% in both outcomes), this asymmetry should not be exclusively attributed to publication bias; it may also reflect true clinical or methodological heterogeneity across intervention types, dosages, and participant characteristics [70]. For subgroup analyses, funnel plots were not generated due to the limited number of studies per subgroup (<10), as visual asymmetry tests are unreliable with small study counts [28].

#### Sensitivity Analysis

Sensitivity analyses were conducted by switching from a random-effects to a fixed-effects model. Under the random-effects model, the pooled effect size for core symptoms was an SMD of 0.26 (95% CI 0.08-0.44; *I*²=77%; *P*=.005), and for cognitive function, it was an SMD of 0.24 (95% CI 0.10-0.39; *I*²=82%; *P*=.001). Under the fixed-effects model, the corresponding values were an SMD of 0.23 (95% CI 0.14-0.31; *I*²=77%; *P*<.001) for core symptoms and an SMD of 0.15 (95% CI 0.09-0.21; *I*²=82%; *P*<.001) for cognitive function. The results did not change meaningfully after the model switch (Figures 5 and 6), indicating that the findings are reasonably robust.

**Figure 5.**
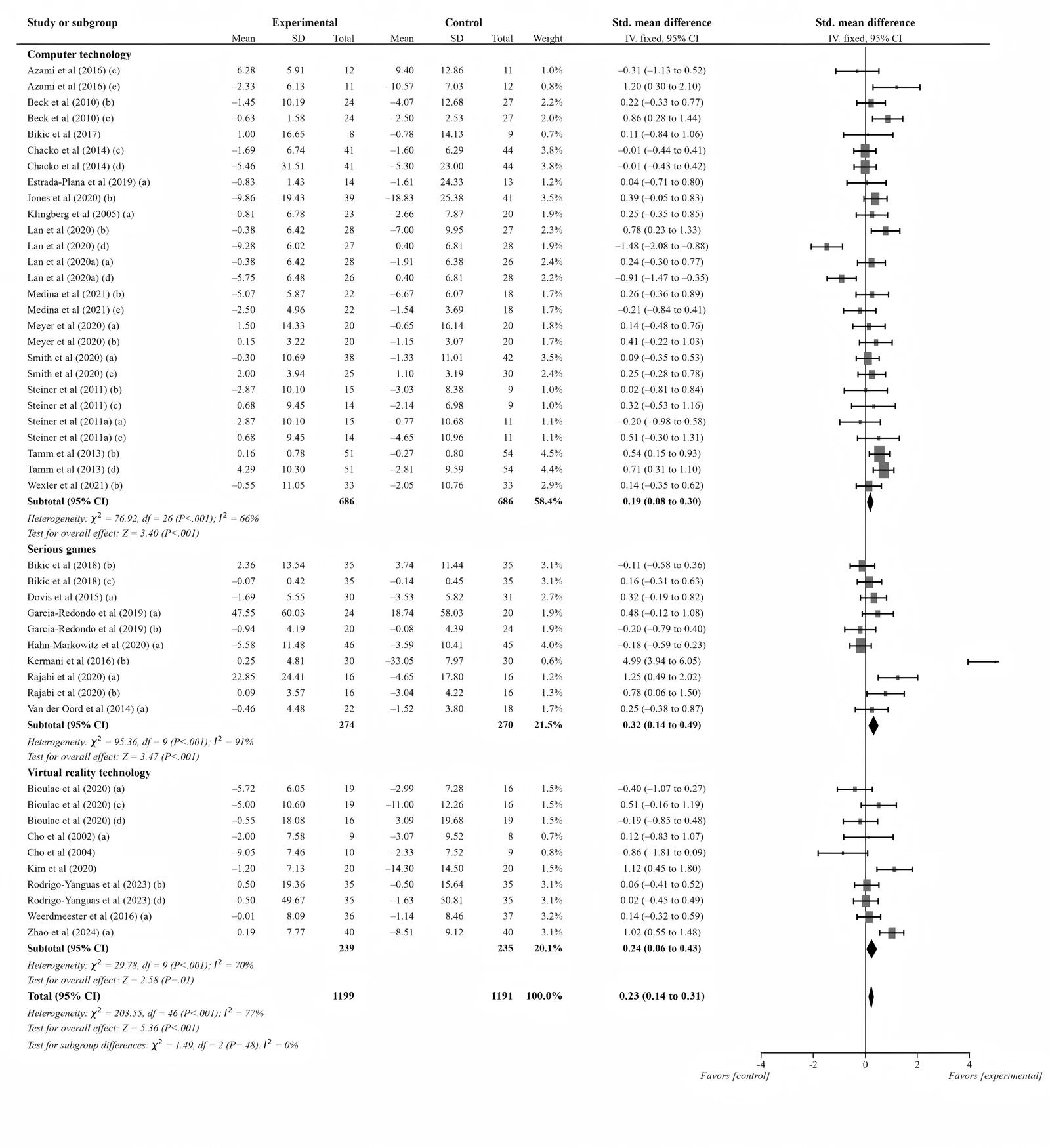
Sensitivity analysis of core symptoms. Lowercase letters in parentheses appended after the study label (eg, (a), (b), (c), (d)) denote different outcome measurement instruments/rating scales used within that study [10,14,17,18,21-23,35,42-47,51-56,58,63-69]. Std: standard.

**Figure 6.**
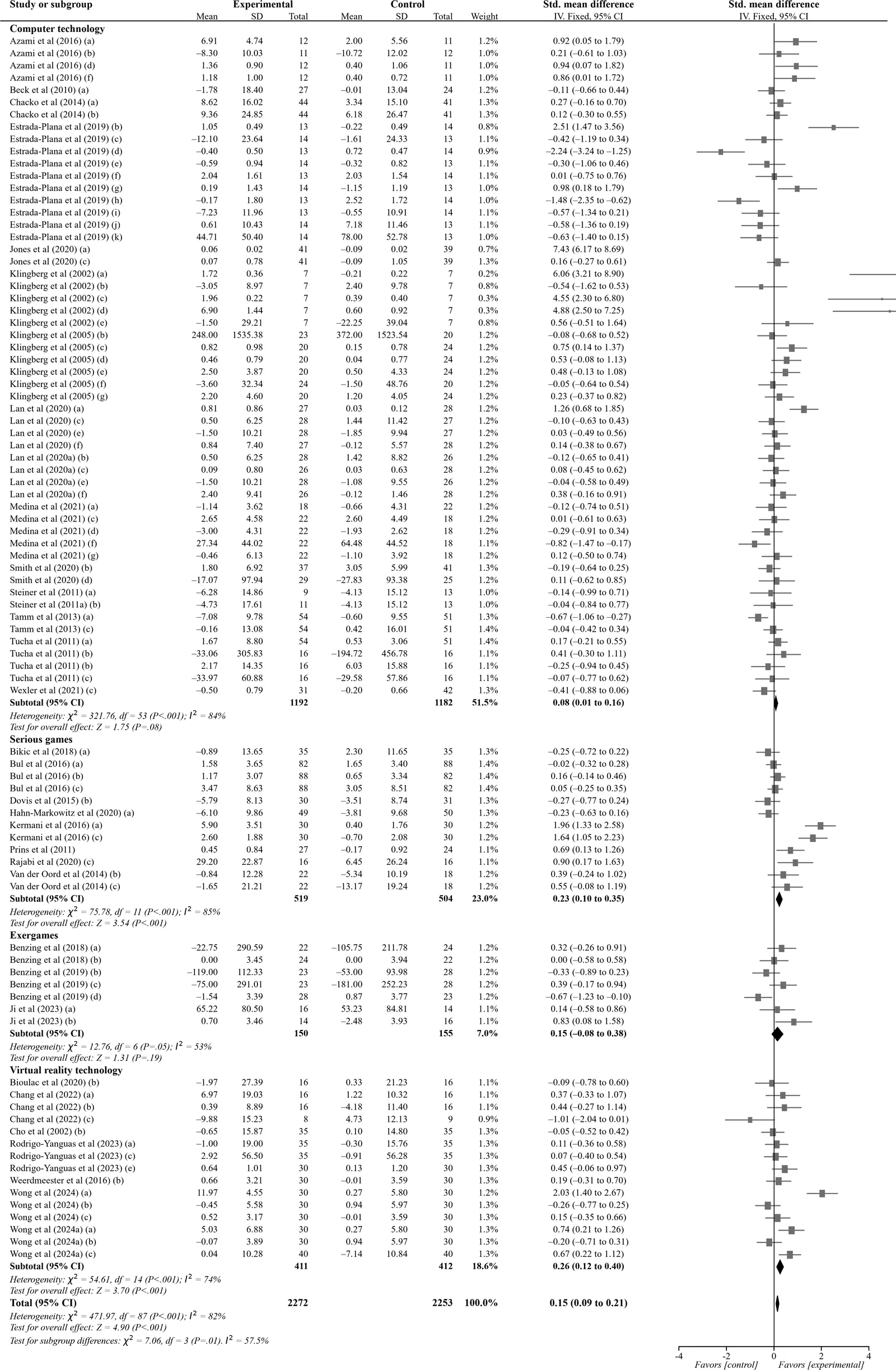
Sensitivity analysis of cognitive function. Lowercase letters in parentheses appended after the study label (eg, (a), (b), (c), (d)) denote different outcome measurement instruments/rating scales used within that study [10,11,17,18,21-23,42,43,45-53,55-65,67,69]. Std: standard.

## Discussion

### Summary of the Findings

This systematic review and meta-analysis included 37 RCTs encompassing 2149 children with ADHD aged 5 to 18 years. The findings demonstrated that digital technology–based interventions yielded small-to-moderate yet statistically significant improvements in both core symptoms (SMD=0.26) and cognitive function (SMD=0.24). These effect sizes align with those reported in previous meta-analyses [15,16]. Among the studied digital intervention types, serious video games produced the most pronounced improvement in core symptoms (SMD=0.68; *P*=.02), whereas both computer-based interventions and serious video games yielded significant improvements in cognitive function. VR interventions exhibited a nonsignificant positive trend, whereas exergame interventions had no significant effects. However, the exergame subgroup included only 3 studies, and its null finding should be interpreted as evidence of insufficient power rather than evidence of no effect. Sensitivity analyses confirmed the robustness of these results.

### Core Symptom Findings

This study found that digital technology–based interventions effectively alleviated core ADHD symptoms, with a small-to-moderate effect size (SMD=0.26). Clinically, this corresponds to mild-to-moderate improvements in both inattention and hyperactivity-impulsivity. Previous meta-analyses have similarly demonstrated that game-based training and computerized cognitive training reduce inattention symptoms [71,72]. These convergent findings underscore the promise of digital tools as clinical adjunctive interventions [32].

Digital interventions, including serious video games and VR, optimize attentional resource allocation by enhancing engagement and providing immediate feedback, thereby improving attention performance [16]. Serious video games, in particular, integrate goal-directed tasks, immediate reinforcement, and immersive motivational design. These features align well with the motivational profile of children with ADHD, who often exhibit low intrinsic motivation and high arousal needs, and promote sustained attention and behavioral inhibition. This supports the view that gamified design enhances training adherence and intervention efficacy [62] and suggests serious video games as the most effective digital modality for improving core symptoms.

In contrast, conventional computer-based training primarily involves repetitive cognitive exercises [58]. Such tasks are less engaging, display fluctuating adherence, and target attention-related cognitive functions in isolation, which may explain why their overall effect on core symptoms was not significant. VR interventions offer greater ecological validity (eg, virtual classrooms); however, most of the included studies had small sample sizes and nonstandardized protocols, and some incorporated mixed-reality technology, potentially diluting the observed effects. This interpretation is consistent with the review by Lin and Chang [31]. Furthermore, concurrent pharmacotherapy may produce additive or interactive effects with digital interventions, influencing the outcomes [69].

The high heterogeneity of core symptom analyses (*I*^2^=77%) was significantly modulated by age, dosage parameters, and control design. Subgroup analyses confirmed age-dependent effects: digital interventions benefited early school-age children (<10 years, SMD=0.36) and late school-age/preadolescent children (10‐11.9 years, SMD=0.15), but not adolescents (≥12 years, SMD=−0.03) [16]. This pattern may stem from adolescents’ complex clinical profiles, mismatched gamified intervention designs, and greater prefrontal plasticity in younger children [73]. Interventions with 21‐ to 45-minute sessions over 5 to 8 weeks were the most stable dosage regimen.

Training frequency showed a counterintuitive trend: low-frequency training (1‐3 sessions/week) yielded significant effects (SMD=0.44) with high heterogeneity (*I*^2^=81%), while high-frequency training (≥4 sessions/week) produced nonsignificant, smaller effects (SMD=0.11) with low heterogeneity (*I*^2^=12%), possibly confounded by varied intervention types. Control conditions exhibited a clear gradient effect: waitlist controls generated a much larger effect (SMD=0.45) than rigorous active controls (SMD=0.17), suggesting substantial placebo and expectancy effects in subjective outcomes [74].

Subjective ratings revealed significant improvements (SMD=0.29; *I^2^*=77%; *P*=.005), whereas objective tests showed no significant changes (SMD=−0.03; *I^2^*=78%; *P*=.87). This discrepancy verifies rater expectancy bias, indicating subjective behavioral improvements do not equate to objective cognitive or behavioral alterations. Subjective and objective measures represent distinct constructs and should not be treated as interchangeable efficacy indicators [25].

In summary, the high heterogeneity of core symptom outcomes reflects systematic clinical variations in intervention characteristics, participant age, dosage schedules, control designs, and measurement tools, rather than random statistical error. Overall pooled effects should be interpreted with subgroup-specific conditional findings to avoid overgeneralization across all children with ADHD. Future stratified studies are required to clarify population-specific responses to digital interventions and improve the clinical interpretability and applicability of relevant results.

### Cognitive Function Findings

Digital interventions demonstrated a significant positive effect on cognitive function (SMD=0.24), consistent with prior evidence. Wong et al [15] reported significant improvements in sustained attention (≈−0.42) and executive function (≈−0.35), and the meta-analysis of Scionti et al [73] of executive function interventions yielded a comparable pooled effect size (SMD≈0.30). Digital training presumably enhances working memory and cognitive flexibility by repeatedly engaging the frontoparietal cognitive network through adaptive-difficulty tasks, consistent with use-dependent neuroplasticity theory.

However, the effects varied by intervention type. In VR interventions, the scenarios’ high complexity and uneven cognitive load may limit the transfer of benefits to contextualized attention, resulting in only modest gains on standardized cognitive measures. In this study, exergame interventions yielded no significant cognitive improvements (SMD=0.17). This null effect may be attributable to dual-task interference: children must simultaneously allocate resources to motor control and cognitive processing, thereby reducing the specificity of cognitive training. This interpretation is consistent with the finding of Benzing and Schmidt [61] that exergames improve reaction time but not core cognitive functions [35]. Nevertheless, given the small number of exergame studies (n=3), this result is exploratory and underpowered; we refrain from concluding that exergames are ineffective. Instead, we suggest that future research should include larger, well-controlled trials with appropriate active control conditions and more comprehensive cognitive assessments to clarify the potential of this hybrid approach.

Pooled cognitive analyses showed high heterogeneity (*I*^2^=82%), which was explored through subgroup analyses. Different digital interventions targeted separate cognitive subdomains: computerized training improved working memory, serious games enhanced executive function, virtual reality optimized attentional control, and somatosensory training facilitated cognitive-motor integration. Differential emphasis on these subdomains across studies led to dispersed effect sizes [25].

Unlike core symptoms, which responded best to medium-term intervention, cognitive benefits were only significant in short-term training (≤4 weeks, SMD=0.33; *P*=.01), while medium-term (SMD=0.14) and long-term (SMD=0.15) training yielded nonsignificant outcomes. This contrast suggests that cognitive improvements depend more on early intervention novelty and intensive neural activation than cumulative training duration. Age disparity further increased heterogeneity: effect sizes differed by 0.12 between younger children and adolescents (SMD=0.19 vs 0.31), and the wide age range (5‐18 years) amplified developmental variability [75].

Heterogeneity also originated from diverse cognitive assessments. Included studies employed over 10 tasks (eg, continuous performance test, N-back, Stroop, Flanker, trail-making test, Cambridge Neuropsychological Test Automated Battery) covering working memory, inhibitory control, cognitive flexibility, and processing speed. These tools vary considerably in psychometric characteristics and sensitivity to training effects. Consistent with core symptom findings, control design and measurement approaches also contributed to cognitive heterogeneity.

In summary, high heterogeneity in cognitive outcomes stems from inconsistencies in intervention targets, training courses, participant age distributions, assessment tools, and study designs [25]. To reduce heterogeneous interference in future meta-analyses, standardized cognitive measures (eg, continuous performance test, Behavior Rating Inventory of Executive Function) and stratified subgroup analyses are recommended to improve the interpretability and credibility of pooled cognitive effects.

### Inconsistent Publication Bias Results

The systematic assessment of publication bias using Begg and Egger tests revealed inconsistent results: the Begg test yielded nonsignificant findings (core symptoms, *P*=.18; cognitive function, *P*=.20), whereas the Egger test indicated potential publication bias (core symptoms, *P*=.02; cognitive function, *P*=.01). Notably, sensitivity analyses by switching from a random-effects model to a fixed-effects model showed that the main conclusions were insensitive to model choice. Nevertheless, while sensitivity analyses supported stable overall result trends, the Egger test revealed potential publication bias, indicating marginally inflated effect size estimates, especially for subjectively assessed core symptoms. Such overestimation may stem from the disproportionate publication of small or positive findings, thereby requiring cautious quantitative interpretation [74].

### Methodological Quality and Its Impact

Methodological quality of 37 RCTs was evaluated using RoB 2.0. Overall, 11 (29.7%) studies had low bias risk, and 26 (70.3%) presented some concerns, while no studies were rated as high risk. Among the 5 assessment domains, outcome measurement showed the highest proportion of methodological issues (n=15, 40%).

Subgroup analyses confirmed that unblinded caregiver assessment was the primary bias source. Caregivers could not be blinded to digital interventions, and their subjective ratings are prone to expectancy bias that inflates behavioral improvements [74]. Awareness of novel digital therapy tends to overestimate core symptom benefits measured by subjective scales (eg, Swanson, Nolan, and Pelham Rating Scale, version IV and Conners’ scales). In contrast, cognitive outcomes were predominantly assessed via objective computerized tasks (eg, Continuous Performance Test, N-back, and Stroop), which are less susceptible to rater bias and provide more robust results [76]. This disparity explains the higher robustness of cognitive outcomes and highlights the need for blinded evaluation or objective indices to reduce detection bias in future trials.

Quality-stratified sensitivity analyses showed that low-bias studies yielded marginally smaller effect sizes for core symptoms (SMD=0.19) and cognitive function (SMD=0.18) than overall estimates, with consistent effect directions and significance, supporting the robustness of primary findings in high-quality subsamples. As “some concerns” in RoB 2.0 do not represent high methodological risk [37], result interpretation should prioritize directional consistency rather than overreliance on individual point estimates.

### Strengths and Limitations

This study has several strengths. It incorporated the most recent digital intervention studies and categorized them by technology type (eg, VR or serious video games), thereby providing a specific evidence base. Additionally, the subjects’ age range was extended to 5 to 18 years, encompassing both childhood and adolescence and addressing the limitation of prior reviews that focused on school-aged children. Furthermore, comprehensive subgroup analyses identified key moderators informing clinical interpretation. This meta-analysis is the first to systematically compare 4 digital intervention modalities and quantify how age, dosage, control type, and measurement tools moderate core symptom and cognitive outcomes.

Several limitations, however, should be acknowledged. First, the limited number of included studies precluded fine-grained subdomain analyses of specific core symptoms (inattention, hyperactivity, and impulsivity) and cognitive subfunctions (eg, inhibitory control, working memory, and cognitive flexibility), potentially masking the targeted effects of different technologies on particular symptoms. Moreover, only 14 of the 37 studies included follow-up data, yielding limited evidence on long-term effects. Second, substantial heterogeneity was observed across the included studies (*I*²>75%), which may have resulted from differences in intervention dosage, age stratification, concurrent medication use, assessment tools, and types of control groups. Additionally, regarding sensitivity analysis, although model switching demonstrated robust findings, leave-one-out sensitivity analysis was not performed. While model switching is an established sensitivity method, leave-one-out analysis would offer additional validation of result stability. We have acknowledged this as a direction for further methodological framework.

### Conclusions

In summary, digital technology–based interventions, as an emerging adjunctive approach, significantly improved core symptoms and cognitive function in children with ADHD. Specifically, the improvements in core symptoms were primarily driven by serious video games, whereas those in cognitive function were mainly attributable to computer-based interventions and serious video games. However, the effects are moderated by age, intervention dosage, control group design, and assessment modality; they are not uniform across all populations or protocols. Future research should address current limitations by optimizing trial designs, elucidating underlying mechanisms, and evaluating long-term effects. Such efforts would accelerate the clinical translation of digital technology–based interventions and provide safe and effective nonpharmacological treatment options for children with ADHD.

## Supplementary material

10.2196/103353 Multimedia Appendix 1 Detailed reasons for full-text exclusions.

10.2196/103353 Multimedia Appendix 2 Detailed study characteristics.

10.2196/103353 Checklist 1 PRISMA checklist.

## Abbreviations

ADHD: attention-deficit/hyperactivity disorder
PICOS: population, intervention, comparison, outcome, study design
PRISMA: Preferred Reporting Items for Systematic Reviews and Meta-Analyses
PROSPERO: International Prospective Register of Systematic Reviews
RCT: randomized controlled trial
RevMan: Review Manager
RoB 2.0: revised Cochrane risk-of-bias tool
SMD: standardized mean difference
VR: virtual reality
