# Supplementary material for: Effect of Digital Technology–Based Interventions on Core Symptoms and Cognitive Function in Children With Attention-Deficit/Hyperactivity Disorder: Systematic Review and Meta-Analysis
Source: J Med Internet Res. 2026 Oct 9;28:e103353. doi: 10.2196/103353 (PMC13652384; doi:10.2196/103353)
Supplement: Supplementary file 1 [file jmir-v28-e103353-s001.docx]

## Multimedia Appendix 1.Search strategy.

| Multimedia Appendix 1. Detailed Reasons for Full-Text Exclusions (n=132) | | | | |
| --- | --- | --- | --- | --- |
| **No.** | **Author (Year)** | **Title** | **DOI** | **Reason for Exclusion** |
| **Review article (systematic review/meta-analysis/narrative review) (n=47)** | | | | |
| 1 | Ameri et al. (2026) | M-Health Interventions to Support Parents of Children with ADHD: A Systematic Review | 10.1007/s10567-026-00583-5 | Review article (systematic review/meta-analysis/narrative review) |
| 2 | Baena et al. (2021) | Effects of Neurofeedback in Children with Attention-Deficit/Hyperactivity Disorder: A Systematic Review | 10.3390/jcm10173797 | Review article (systematic review/meta-analysis/narrative review) |
| 3 | Basciano et al. (2025) | Multiple Ways in Which Video Games Make Education Inclusive: A Systematic Review of Cognitive Enhancement for Neurodivergent Learners | 10.3390/mti9060056 | Review article (systematic review/meta-analysis/narrative review) |
| 4 | Bashiri et al. (2017) | The opportunities of virtual reality in the rehabilitation of children with attention deficit hyperactivity disorder: a literature review | 10.3345/kjp.2017.60.11.337 | Review article (systematic review/meta-analysis/narrative review) |
| 5 | Cao et al. (2026) | Virtual reality interventions for attention deficit/hyperactivity disorder: a systematic review | 10.1038/s41746-026-02505-9 | Review article (systematic review/meta-analysis/narrative review) |
| 6 | Caselles-Pina et al. (2023) | Adherence, frequency, and long-term follow-up of video game-based treatments in patients with attention-deficit/hyperactivity disorder: A systematic review | 10.1002/brb3.3265 | Review article (systematic review/meta-analysis/narrative review) |
| 7 | Ceyhan & Akyurek (2026) | Technology-Based Interventions for Children and Adolescents with ADHD: A Systematic Review and Meta-Analysis | 10.1080/01942638.2026.2689070 | Review article (systematic review/meta-analysis/narrative review) |
| 8 | Chen et al. (2022) | Which Factor Is More Relevant to the Effectiveness of the Cognitive Intervention? A Meta-Analysis of Randomized Controlled Trials of Cognitive Training on Symptoms and Executive Function Behaviors of Children With Attention Deficit Hyperactivity Disorder | 10.3389/fpsyg.2021.810298 | Review article (systematic review/meta-analysis/narrative review) |
| 9 | Cho & Talboys (2024) | Trends in South Korean Medical Device Development for Attention-Deficit/Hyperactivity Disorder and Autism Spectrum Disorder: Narrative Review | 10.2196/60399 | Review article (systematic review/meta-analysis/narrative review) |
| 10 | Chung et al. (2026) | Systematic review of virtual reality therapy in the treatment of visual dysfunction in children with ADHD and dyslexia | 10.1016/j.neubiorev.2026.106723 | Review article (systematic review/meta-analysis/narrative review) |
| 11 | Cibrian et al. (2024) | Digital assessments for children and adolescents with ADHD: a scoping review | 10.3389/fdgth.2024.1440701 | Review article (systematic review/meta-analysis/narrative review) |
| 12 | Corrigan et al. (2023) | Immersive virtual reality for improving cognitive deficits in children with ADHD: a systematic review and meta-analysis | 10.1007/s10055-023-00768-1 | Review article (systematic review/meta-analysis/narrative review) |
| 13 | Cortese et al. (2015) | Cognitive training for attention-deficit/hyperactivity disorder: meta-analysis of clinical and neuropsychological outcomes from randomized controlled trials | 10.1016/j.jaac.2014.12.010 | Review article (systematic review/meta-analysis/narrative review) |
| 14 | Doulou et al. (2025) | Managing ADHD Symptoms in Children Through the Use of Various Technology-Driven Serious Games: A Systematic Review | 10.3390/mti9010008 | Review article (systematic review/meta-analysis/narrative review) |
| 15 | Doulou et al. (2025) | Virtual reality programs targeting executive functions and social cognition evaluation and/or rehabilitation in children with ADHD or ASD-A narrative review | 10.3389/fpsyg.2025.1583052 | Review article (systematic review/meta-analysis/narrative review) |
| 16 | Doulou et al. (2026) | Technological Devices for Developing Working Memory in Children with ADHD: A Systematic Review | 10.3390/psychiatryint7030104 | Review article (systematic review/meta-analysis/narrative review) |
| 17 | Elbe et al. (2023) | Computerized Cognitive Interventions for Adults With ADHD: A Systematic Review and Meta-Analysis | 10.1037/neu0000890 | Review article (systematic review/meta-analysis/narrative review) |
| 18 | Gabarron et al. (2025) | Evaluating the evidence: a systematic review of reviews of the effectiveness and safety of digital interventions for ADHD | 10.1186/s12888-025-06825-0 | Review article (systematic review/meta-analysis/narrative review) |
| 19 | González-Bracamonte et al. (2023) | Efficacy of virtual reality in the evaluation and treatment of adhd: a systematic review of the literature | 10.15332/22563067.9375 | Review article (systematic review/meta-analysis/narrative review) |
| 20 | Hamada et al. (2025) | Enhancing effects of exercise and neurofeedback: A systematic review and meta-analysis of computer game-based interventions for pediatric ADHD | 10.1016/j.psychres.2025.116447 | Review article (systematic review/meta-analysis/narrative review) |
| 21 | Hao et al. (2026) | Multimodal Digital Therapeutics Enhanced by Task Design and AI for Attention-Deficit/Hyperactivity Disorder Core Symptoms and Executive Functions in Children and Adolescents: Systematic Review and Network Meta-Analysis of Randomized Controlled Trials | 10.2196/95043 | Review article (systematic review/meta-analysis/narrative review) |
| 22 | Hassan Awaji et al. (2026) | Mapping the Mind: A Network Meta-Analysis of Mindfulness and Traditional and Digital Interventions for Cognitive and Behavioral Enhancement in Children With Attention-Deficit/Hyperactivity Disorder (ADHD) | 10.7759/cureus.102453 | Review article (systematic review/meta-analysis/narrative review) |
| 23 | He et al. (2023) | Meta-analysis of the efficacy of digital therapies in children with attention-deficit hyperactivity disorder | 10.3389/fpsyt.2023.1054831 | Review article (systematic review/meta-analysis/narrative review) |
| 24 | Khan et al. (2019) | The Effectiveness of Web-Based Interventions Delivered to Children and Young People With Neurodevelopmental Disorders: Systematic Review and Meta-Analysis | 10.2196/13478 | Review article (systematic review/meta-analysis/narrative review) |
| 25 | Konaç et al. (2024) | Enhancing Executive Function Skills in Children With Attention-Deficit/Hyperactivity Disorder via Immersive Virtual Reality Interventions: Scoping Review | 10.2196/57225 | Review article (systematic review/meta-analysis/narrative review) |
| 26 | Lakes et al. (2022) | Digital health interventions for youth with ADHD: A mapping review | 10.1016/j.chbr.2022.100174 | Review article (systematic review/meta-analysis/narrative review) |
| 27 | Lee et al. (2022) | Can Neurocognitive Outcomes Assist Measurement-Based Care for Children with Attention-Deficit/Hyperactivity Disorder? A Systematic Review and Meta-Analyses of the Relationships Among the Changes in Neurocognitive Functions and Clinical Outcomes of Attention-Deficit/Hyperactivity Disorder in Pharmacological and Cognitive Training Interventions | 10.1089/cap.2022.0028 | Review article (systematic review/meta-analysis/narrative review) |
| 28 | Lee et al. (2025) | Effectiveness of game-based digital intervention for attention-deficit hyperactivity disorder in children and adolescents: a systematic review and meta-analysis using Beard and Wilson's conceptualization of perception in experiential learning | 10.1007/s00787-025-02788-5 | Review article (systematic review/meta-analysis/narrative review) |
| 29 | Lin & Chang (2025) | Effectiveness of Serious Games as Digital Therapeutics for Enhancing the Abilities of Children With Attention-Deficit/Hyperactivity Disorder (ADHD): Systematic Literature Review | 10.2196/60937 | Review article (systematic review/meta-analysis/narrative review) |
| 30 | Mao et al. (2025) | Efficacy of game-based interventions on cognitive performance in children and adolescents with attention-deficit/hyperactivity disorder: a systematic review and meta-analysis | 10.1186/s12888-025-07607-4 | Review article (systematic review/meta-analysis/narrative review) |
| 31 | Mei et al. (2026) | Effectiveness of different digital interventions on symptoms for children and adolescents with attention-deficit/hyperactivity disorder: a network meta-analysis | 10.3389/fpsyt.2026.1747368 | Review article (systematic review/meta-analysis/narrative review) |
| 32 | Murray et al. (2025) | A Narrative Review to Identify Promising Approaches for Digital Health Interventions to Support Emotion Regulation for Adolescents With Attention-Deficit/Hyperactivity Disorder | 10.2196/56066 | Review article (systematic review/meta-analysis/narrative review) |
| 33 | Parsons et al. (2019) | A Comparison of Virtual Reality Classroom Continuous Performance Tests to Traditional Continuous Performance Tests in Delineating ADHD: a Meta-Analysis | 10.1007/s11065-019-09407-6 | Review article (systematic review/meta-analysis/narrative review) |
| 34 | Peñuelas-Calvo et al. (2022) | Video games for the assessment and treatment of attention-deficit/hyperactivity disorder: a systematic review | 10.1007/s00787-020-01557-w | Review article (systematic review/meta-analysis/narrative review) |
| 35 | Poissant et al. (2019) | Behavioral and Cognitive Impacts of Mindfulness-Based Interventions on Adults with Attention-Deficit Hyperactivity Disorder: A Systematic Review | 10.1155/2019/5682050 | Review article (systematic review/meta-analysis/narrative review) |
| 36 | Powell et al. (2018) | What is the level of evidence for the use of currently available technologies in facilitating the self-management of difficulties associated with ADHD in children and young people? A systematic review | 10.1007/s00787-017-1092-x | Review article (systematic review/meta-analysis/narrative review) |
| 37 | Pradana et al. (2026) | The effects of exergaming on executive functions in children with ADHD: a systematic review and meta-analysis of randomised controlled trials | 10.1080/21642850.2026.2614157 | Review article (systematic review/meta-analysis/narrative review) |
| 38 | Robledo-Castro et al. (2023) | Effect of Cognitive Training Programs Based on Computer Systems on Executive Functions in Children With ADHD: A Systematic Review | 10.1177/10870547231187164 | Review article (systematic review/meta-analysis/narrative review) |
| 39 | Romero-Ayuso et al. (2021) | Effectiveness of Virtual Reality-Based Interventions for Children and Adolescents with ADHD: A Systematic Review and Meta-Analysis | 10.3390/children8020070 | Review article (systematic review/meta-analysis/narrative review) |
| 40 | Schofield et al. (2026) | Digital health technologies for adults with ADHD: a scoping review | 10.3389/fdgth.2026.1746732 | Review article (systematic review/meta-analysis/narrative review) |
| 41 | Sun et al. (2025) | Effects of virtual reality-based exercise intervention in young people with attention-deficit/ hyperactivity disorder: a systematic review | 10.1186/s12984-025-01671-3 | Review article (systematic review/meta-analysis/narrative review) |
| 42 | Venturo-Conerly et al. (2022) | Effectiveness of Youth Psychotherapy Delivered Remotely: A Meta-Analysis | 10.1037/amp0000816 | Review article (systematic review/meta-analysis/narrative review) |
| 43 | Wong et al. (2023) | Effectiveness of Technology-Based Interventions for School-Age Children With Attention-Deficit/Hyperactivity Disorder: Systematic Review and Meta-Analysis of Randomized Controlled Trials | 10.2196/51459 | Review article (systematic review/meta-analysis/narrative review) |
| 44 | Yan et al. (2026) | The effects of computerized executive function training on the youth with ADHD: A systematic review and meta-analysis | 10.1016/j.jad.2025.120730 | Review article (systematic review/meta-analysis/narrative review) |
| 45 | Zhang et al. (2024) | A Study of the Effects of Virtual Reality-Based Sports Games on Improving Executive and Cognitive Functions in Minors with ADHD-A Meta-Analysis of Randomized Controlled Trials | 10.3390/bs14121141 | Review article (systematic review/meta-analysis/narrative review) |
| 46 | Zhong et al. (2025) | Neurofeedback training for executive function in ADHD children: a systematic review and meta-analysis | 10.1038/s41598-025-94242-4 | Review article (systematic review/meta-analysis/narrative review) |
| 47 | Zou et al. (2025) | The effect of cognitive training on children with attention deficit and hyperactivity disorder: A meta-analysis | 10.1080/21622965.2024.2305874 | Review article (systematic review/meta-analysis/narrative review) |
| **Study protocol (no results reported) (n=35)** | | | | |
| 48 | Arenas et al. (2024) | Effects of active breaks on educational achievement in children with and without ADHD: study protocol and rationale of the Break4Brain project | 10.3389/fpsyg.2024.1451731 | Study protocol (no results reported) |
| 49 | Badia-Aguarón et al. (2024) | Combining mindfulness and cognitive training in children with attention deficit hyperactivity disorder: study protocol of a pilot randomized controlled trial (the NeuroMind study) | 10.3389/fpsyg.2024.1291198 | Study protocol (no results reported) |
| 50 | Bikic et al. (2015) | Cognitive computer training in children with attention deficit hyperactivity disorder (ADHD) versus no intervention: study protocol for a randomized controlled trial | 10.1186/s13063-015-0975-8 | Study protocol (no results reported) |
| 51 | Bikic et al. (2021) | Organizational skills training for children with ADHD: study protocol for a randomized, controlled trial | 10.1186/s13063-021-05499-9 | Study protocol (no results reported) |
| 52 | Blasco-Fontecilla et al. (2026) | Measuring Accuracy (Classification Probabilities, Positive, and Negative Predictive Values) of Executive Function Electroencephalogram Metrics in Attention-Deficit/Hyperactivity Disorder Diagnosis: Protocol for and Perspectives From the SINCRONIA Study | 10.2196/79150 | Study protocol (no results reported) |
| 53 | Blume et al. (2017) | NIRS-based neurofeedback training in a virtual reality classroom for children with attention-deficit/hyperactivity disorder: study protocol for a randomized controlled trial | 10.1186/s13063-016-1769-3 | Study protocol (no results reported) |
| 54 | Calderon et al. (2019) | Improving neurodevelopmental outcomes in children with congenital heart disease: protocol for a randomised controlled trial of working memory training | 10.1136/bmjopen-2018-023304 | Study protocol (no results reported) |
| 55 | Chan et al. (2018) | Effects of a mindfulness-based intervention (MYmind) for children with ADHD and their parents: protocol for a randomised controlled trial | 10.1136/bmjopen-2018-022514 | Study protocol (no results reported) |
| 56 | Dyresen et al. (2024) | Executive control training for adolescents with ADHD: Study protocol for a randomised controlled trial | 10.1016/j.cct.2023.107404 | Study protocol (no results reported) |
| 57 | Geissler et al. (2018) | Individualised short-term therapy for adolescents impaired by attention-deficit/hyperactivity disorder despite previous routine care treatment (ESCAadol)-Study protocol of a randomised controlled trial within the consortium ESCAlife | 10.1186/s13063-018-2635-2 | Study protocol (no results reported) |
| 58 | González-Pérez et al. (2025) | Study protocol for a randomized controlled clinical trial of a multifaceted cognitive training program using video games in childhood cancer survivors | 10.1371/journal.pone.0314118 | Study protocol (no results reported) |
| 59 | Goodwin et al. (2016) | Attention training for infants at familial risk of ADHD (INTERSTAARS): study protocol for a randomised controlled trial | 10.1186/s13063-016-1727-0 | Study protocol (no results reported) |
| 60 | Herrera et al. (2024) | Digital health intervention for children with ADHD to improve mental health intervention, patient experiences, and outcomes: a study protocol | 10.1186/s44247-024-00134-4 | Study protocol (no results reported) |
| 61 | Hogue et al. (2016) | Changing Academic Support in the Home for Adolescents With Attention-Deficit/Hyperactivity Disorder: A Family-Based Clinical Protocol for Improving School Performance | 10.1016/j.cbpra.2014.07.002 | Study protocol (no results reported) |
| 62 | Jostrup et al. (2024) | Effects of Auditory and Visual White Noise on Oculomotor Inhibition in Children With Attention-Deficit/Hyperactivity Disorder: Protocol for a Crossover Study | 10.2196/56388 | Study protocol (no results reported) |
| 63 | Lo et al. (2016) | The effect of a family-based mindfulness intervention on children with attention deficit and hyperactivity symptoms and their parents: design and rationale for a randomized, controlled clinical trial (Study protocol) | 10.1186/s12888-016-0773-1 | Study protocol (no results reported) |
| 64 | Martin-Moratinos et al. (2024) | Effectiveness of a Serious Video Game (MOON) for Attention Deficit Hyperactivity Disorder: Protocol for a Randomized Clinical Trial | 10.2196/53191 | Study protocol (no results reported) |
| 65 | Marx et al. (2019) | Perceptual timing in children with attention-deficit/hyperactivity disorder (ADHD) as measured by computerbased experiments versus real-life tasks: protocol for a cross-sectional experimental study in an ambulatory setting | 10.1136/bmjopen-2018-027651 | Study protocol (no results reported) |
| 66 | Mauche et al. (2024) | Treatment of adult attention-deficit hyperactivity disorder (ADHD) with transcranial direct current stimulation (tDCS): study protocol for a parallel, randomized, double-blinded, sham-controlled, multicenter trial (Stim-ADHD) | 10.1007/s00406-023-01652-4 | Study protocol (no results reported) |
| 67 | Mayer et al. (2018) | Bright light therapy versus physical exercise to prevent co-morbid depression and obesity in adolescents and young adults with attention-deficit / hyperactivity disorder: study protocol for a randomized controlled trial | 10.1186/s13063-017-2426-1 | Study protocol (no results reported) |
| 68 | McKay et al. (2019) | Training attention in children with acquired brain injury: a study protocol of a randomised controlled trial of the TALI attention training programme | 10.1136/bmjopen-2019-032619 | Study protocol (no results reported) |
| 69 | Meller et al. (2026) | High-Intensity Virtual Reality Exergaming for Adolescents With Attention-Deficit/Hyperactivity Disorder: Protocol for a Randomized Clinical Trial | 10.2196/94797 | Study protocol (no results reported) |
| 70 | Pérez-Rodríguez et al. (2025) | ECogFun-virtual reality program for improving planning and organization skills in daily life among children and adolescents with ADHD: A study protocol for a randomized controlled trial | 10.1016/j.ridd.2025.105075 | Study protocol (no results reported) |
| 71 | Richmond et al. (2022) | Digital cognitive training in children with attention-deficit/hyperactivity disorder: a study protocol of a randomised controlled trial | 10.1136/bmjopen-2021-055385 | Study protocol (no results reported) |
| 72 | Rivas-García et al. (2026) | Feasibility of "DiverAcción": A Web-Based Telerehabilitation System for Executive Functions Training in Children and Adolescents with ADHD-Longitudinal Study Protocol | 10.3390/healthcare14030323 | Study protocol (no results reported) |
| 73 | Salzmann et al. (2026) | A Metacognitive Intervention for Children and Adolescents in Neuropediatric Care (Mio-Training): Protocol for a Randomized Controlled Trial | 10.2196/95139 | Study protocol (no results reported) |
| 74 | Sciberras et al. (2017) | Does a brief, behavioural intervention, delivered by paediatricians or psychologists improve sleep problems for children with ADHD? Protocol for a cluster-randomised, translational trial | 10.1136/bmjopen-2016-014158 | Study protocol (no results reported) |
| 75 | Siebelink et al. (2018) | Mindfulness for children with ADHD and Mindful Parenting (MindChamp): Protocol of a randomised controlled trial comparing a family Mindfulness-Based Intervention as an add-on to care-as-usual with care-as-usual only | 10.1186/s12888-018-1811-y | Study protocol (no results reported) |
| 76 | Sun et al. (2022) | Effect of game-based high-intensity interval training program on the executive function of children with ADHD: Protocol of a randomized controlled trial | 10.1371/journal.pone.0272121 | Study protocol (no results reported) |
| 77 | Wong & Qin (2023) | Effectiveness of Social Virtual Reality Training in Enhancing Social Interaction Skills in Children With Attention-Deficit/Hyperactivity Disorder: Protocol for a Three-Arm Pilot Randomized Controlled Trial | 10.2196/48208 | Study protocol (no results reported) |
| 78 | Yan et al. (2018) | Effects of neurofeedback versus methylphenidate for the treatment of attention-deficit/hyperactivity disorder protocol for a systematic review and meta-analysis of head-to-head trials | 10.1097/md.0000000000012623 | Study protocol (no results reported) |
| 79 | Zhang et al. (2025) | A randomized controlled trial for evaluating the effects of an online mindfulness-based intervention for parents of children with attention-deficit/hyperactivity disorder: a study protocol | 10.1186/s40359-025-02929-0 | Study protocol (no results reported) |
| 80 | Zhang et al. (2026) | Effects of open- versus closed-skill exercise combined with mindfulness training on inhibitory control in children with ADHD: protocol for a three-arm randomised controlled trial with fMRI | 10.1136/bmjopen-2026-116849 | Study protocol (no results reported) |
| 81 | Zhao et al. (2026) | Cross-training digital therapeutics for pediatric ADHD: study protocol for a randomized controlled trial | 10.1186/s13063-026-09775-4 | Study protocol (no results reported) |
| 82 | Zheng et al. (2024) | Study protocol of a randomised controlled trial of the effects of near-infrared spectroscopy neurofeedback training coupled with virtual reality technology in children with ADHD | 10.1136/bmjopen-2024-093183 | Study protocol (no results reported) |
| **Longitudinal/observational study (not RCT) (n=2)** | | | | |
| 83 | Cheng et al. (2026) | Effects of computerized cognitive training on brain function in children with ADHD: A longitudinal neuroimaging study based on fALFF | 10.1016/j.bbr.2025.115895 | Longitudinal/observational study (not RCT) |
| 84 | Soares et al. (2022) | Is Screen Time Throughout Adolescence Related to ADHD? Findings from 1993 Pelotas (Brazil) Birth Cohort Study | 10.1177/1087054721997555 | Longitudinal/observational study (not RCT) |
| **Cross-sectional study (not intervention trial) (n=2)** | | | | |
| 85 | Altun Varmış et al. (2026) | Problematic digital use in adolescents with ADHD: impulsivity, obsessive-compulsive symptoms, and perceived cognitive flexibility | 10.1186/s40359-026-05200-2 | Cross-sectional study (not intervention trial) |
| 86 | Park et al. (2026) | A Digital Tool for Assessing the Distinct Effects of Depression, Anxiety, and Attention-Deficit/Hyperactivity Disorder (ADHD) on Children's Emotional Cognitive Bias: Cross-Sectional Study | 10.2196/86286 | Cross-sectional study (not intervention trial) |
| **Study population: adults (18+ years) (n=19)** | | | | |
| 87 | BarNir et al. (2023) | Evidence for Overlapping Visual Processing Difficulties in Adult ADHD and Visual Stress | 10.1177/00315125231192809 | Study population: adults (18+ years) |
| 88 | Barth et al. (2021) | A randomized-controlled neurofeedback trial in adult attention-deficit/hyperactivity disorder | 10.1038/s41598-021-95928-1 | Study population: adults (18+ years) |
| 89 | Cortez-Carbonell & Ceric (2017) | Emotion recognition of facial expressions in adults with attention deficit hyperactivity disorder | 10.1080/02109395.2016.1268387 | Study population: adults (18+ years) |
| 90 | Cowley et al. (2016) | Computer Enabled Neuroplasticity Treatment: A Clinical Trial of a Novel Design for Neurofeedback Therapy in Adult ADHD | 10.3389/fnhum.2016.00205 | Study population: adults (18+ years) |
| 91 | da Costa et al. (2026) | Towards ecological validity when assessing ADHD symptoms: Patterns in automatically collected, real-world PC activity data | 10.1016/j.ijhcs.2025.103724 | Study population: adults (18+ years) |
| 92 | Delisle & Braun (2011) | A Context for Normalizing Impulsiveness at Work for Adults with Attention Deficit/Hyperactivity Disorder (Combined Type) | 10.1093/arclin/acr043 | Study population: adults (18+ years) |
| 93 | Dentz et al. (2020) | Working Memory Training for Adults With ADHD | 10.1177/1087054717723987 | Study population: adults (18+ years) |
| 94 | Hudak et al. (2017) | Near-Infrared Spectroscopy-Based Frontal Lobe Neurofeedback Integrated in Virtual Reality Modulates Brain and Behavior in Highly Impulsive Adults | 10.3389/fnhum.2017.00425 | Study population: adults (18+ years) |
| 95 | Jylkkä et al. (2023) | Assessment of goal-directed behavior and prospective memory in adult ADHD with an online 3D videogame simulating everyday tasks | 10.1038/s41598-023-36351-6 | Study population: adults (18+ years) |
| 96 | Lintas et al. (2025) | Boosting Working Memory in ADHD: Adaptive Dual N-Back Training Enhances WAIS-IV Performance, but Yields Mixed Corsi Outcomes | 10.3390/brainsci15090998 | Study population: adults (18+ years) |
| 97 | Liu et al. (2016) | EEG alpha power during maintenance of information in working memory in adults with ADHD and its plasticity due to working memory training: A randomized controlled trial | 10.1016/j.clinph.2015.10.032 | Study population: adults (18+ years) |
| 98 | Marcelle et al. (2018) | Cogmed Working Memory Training Presents Unique Implementation Challenges in Adults With ADHD | 10.3389/fpsyt.2018.00388 | Study population: adults (18+ years) |
| 99 | Nasri et al. (2023) | Internet delivered cognitive behavioral therapy for adults with ADHD - A randomized controlled trial | 10.1016/j.invent.2023.100636 | Study population: adults (18+ years) |
| 100 | Osman et al. (2025) | Real-world effectiveness of a widely available digital health program in adults reporting a lifetime diagnosis of ADHD | 10.1038/s44184-025-00157-3 | Study population: adults (18+ years) |
| 101 | Stern et al. (2016) | The Efficacy of Computerized Cognitive Training in Adults With ADHD: A Randomized Controlled Trial | 10.1177/1087054714529815 | Study population: adults (18+ years) |
| 102 | Stern et al. (2023) | Near and Far Transfer Effects of Computerized Progressive Attention Training (CPAT) Versus Mindfulness Based Stress Reduction (MBSR) Practice Among Adults With ADHD | 10.1177/10870547231155877 | Study population: adults (18+ years) |
| 103 | Tolonen et al. (2024) | Working memory related functional connectivity in adult ADHD and its amenability to training: A randomized controlled trial | 10.1016/j.nicl.2024.103696 | Study population: adults (18+ years) |
| 104 | Virta et al. (2010) | Short cognitive behavioral therapy and cognitive training for adults with ADHD - a randomized controlled pilot study | 10.2147/ndt.s11743 | Study population: adults (18+ years) |
| 105 | Zilverstand et al. (2017) | fMRI Neurofeedback Training for Increasing Anterior Cingulate Cortex Activation in Adult Attention Deficit Hyperactivity Disorder. An Exploratory Randomized, Single-Blinded Study | 10.1371/journal.pone.0170795 | Study population: adults (18+ years) |
| **Study population: typically developing children (n=9)** | | | | |
| 106 | Anguera et al. (2016) | Characterizing cognitive control abilities in children with 16p11.2 deletion using adaptive 'video game' technology: a pilot study | 10.1038/tp.2016.178 | Study population: typically developing children |
| 107 | Anguera et al. (2023) | Enhancing attention in children using an integrated cognitive-physical videogame: A pilot study | 10.1038/s41746-023-00812-z | Study population: typically developing children |
| 108 | Bush et al. (2015) | Feasibility and validity of the structured attention module among economically disadvantaged preschool-age children | 10.1080/09297049.2014.886676 | Study population: typically developing children |
| 109 | Cubillo et al. (2023) | Intra-individual variability in task performance after cognitive training is associated with long-term outcomes in children | 10.1111/desc.13252 | Study population: typically developing children |
| 110 | Flynn & Colon (2016) | Solitary Active Videogame Play Improves Executive Functioning More Than Collaborative Play for Children with Special Needs | 10.1089/g4h.2016.0053 | Study population: typically developing children |
| 111 | Giovannetti et al. (2020) | Impact of an Individualized Cognitive Training Intervention in Preschoolers from Poor Homes | 10.3390/ijerph17082912 | Study population: typically developing children |
| 112 | Graham & Benninger (2016) | Parental Perceptions of the Efficacy of Cogmed Working Memory Training | 10.1080/21622965.2016.1167505 | Study population: typically developing children |
| 113 | Kirk et al. (2017) | Impact of Attention Training on Academic Achievement, Executive Functioning, and Behavior: A Randomized Controlled Trial | 10.1352/1944-7558-122.2.97 | Study population: typically developing children |
| 114 | Roberts et al. (2016) | Academic Outcomes 2 Years After Working Memory Training for Children With Low Working Memory: A Randomized Clinical Trial | 10.1001/jamapediatrics.2015.4568 | Study population: typically developing children |
| **Study population: other clinical conditions (not ADHD) (n=11)** | | | | |
| 115 | Au et al. (2014) | A feasibility trial of Cogmed working memory training in fragile X syndrome | 10.3233/pge-14098 | Study population: other clinical conditions (not ADHD) |
| 116 | Calub et al. (2022) | Working Memory Training in Youth With Autism, Fragile X, and Intellectual Disability: A Pilot Study: AJMR | 10.1352/1944-7558-127.5.369 | Study population: other clinical conditions (not ADHD) |
| 117 | Dashi & Strigas (2026) | The effects of virtual reality and sports activities on improving learning disabilities | 10.7752/jpes.2026.02020 | Study population: other clinical conditions (not ADHD) |
| 118 | Hardy et al. (2011) | Computerized cognitive training in survivors of childhood cancer: a pilot study | 10.1177/1043454210377178 | Study population: other clinical conditions (not ADHD) |
| 119 | Kerns et al. (2010) | Investigating the efficacy of an attention training programme in children with foetal alcohol spectrum disorder | 10.3109/17518423.2010.511421 | Study population: other clinical conditions (not ADHD) |
| 120 | Ko et al. (2021) | Effect of Anodal Transcranial Direct Current Stimulation Combined With Cognitive Training for Improving Cognition and Language Among Children With Cerebral Palsy With Cognitive Impairment: A Pilot, Randomized, Controlled, Double-Blind, and Clinical Trial | 10.3389/fped.2021.713792 | Study population: other clinical conditions (not ADHD) |
| 121 | Ottersen & Grill (2015) | Benefits of extending and adjusting the level of difficulty on computerized cognitive training for children with intellectual disabilities | 10.3389/fpsyg.2015.01233 | Study population: other clinical conditions (not ADHD) |
| 122 | Peyre et al. (2018) | [Computerized oculomotor training in dyslexia: A randomized, crossover clinical trial in pediatric population] | 10.1016/j.encep.2017.03.004 | Study population: other clinical conditions (not ADHD) |
| 123 | Roording-Ragetlie et al. (2023) | Working memory training in children with neurodevelopmental disorders and intellectual disabilities, the role of coaching: A double-blind randomised controlled trial | 10.1111/jir.13047 | Study population: other clinical conditions (not ADHD) |
| 124 | Saard et al. (2017) | Neurorehabilitation with FORAMENRehab for attention impairment in children with epilepsy | 10.1016/j.yebeh.2016.12.030 | Study population: other clinical conditions (not ADHD) |
| 125 | Weckstein et al. (2017) | A Retrospective Chart Analysis with Follow-Up of Cogmed Working Memory Training in Children and Adolescents with Autism Spectrum Disorder | 10.12659/msmbr.904930 | Study population: other clinical conditions (not ADHD) |
| **Study population: preschool children (<6 years) / infants (n=3)** | | | | |
| 126 | Capodieci et al. (2018) | Effects of a working memory training program in preschoolers with symptoms of attention-deficit/hyperactivity disorder | 10.1080/13803395.2017.1307946 | Study population: preschool children (<6 years) / infants |
| 127 | Goodwin et al. (2026) | Randomised Controlled Trial of Gaze-Based Attention Training Intervention for Infants With a Family History of Attention Deficit Hyperactivity Disorder: Follow-up Outcomes at 2 and 3 Years | 10.1177/10870547261428660 | Study population: preschool children (<6 years) / infants |
| 128 | Zheng & Shum (2025) | Brief Report: A Randomized Controlled Trial of a Digital Working Memory Intervention for Preschoolers Displaying ADHD Symptoms | 10.1007/s10803-023-06213-1 | Study population: preschool children (<6 years) / infants |
| **Study population: mixed samples (ADHD + non-ADHD not separable) (n=4)** | | | | |
| 129 | Areces et al. (2018) | Analysis of cognitive and attentional profiles in children with and without ADHD using an innovative virtual reality tool | 10.1371/journal.pone.0201039 | Study population: mixed samples (ADHD + non-ADHD not separable) |
| 130 | Dehnabaei et al. (2024) | Computerized cognitive training to improve executive functions and driving skills of adolescents with and without symptoms of attention-deficit/hyperactivity disorder | 10.1016/j.trf.2024.06.019 | Study population: mixed samples (ADHD + non-ADHD not separable) |
| 131 | Johnstone et al. (2012) | Neurocognitive training for children with and without AD/HD | 10.1007/s12402-011-0069-8 | Study population: mixed samples (ADHD + non-ADHD not separable) |
| 132 | Kiani et al. (2024) | Examining emotion regulation using a distraction and reappraisal task in children and adolescents with and without ADHD | 10.1007/s12144-023-04766-z | Study population: mixed samples (ADHD + non-ADHD not separable) |
