# Supplementary material for: Effect of Digital Technology–Based Interventions on Core Symptoms and Cognitive Function in Children With Attention-Deficit/Hyperactivity Disorder: Systematic Review and Meta-Analysis
Source: J Med Internet Res. 2026 Oct 9;28:e103353. doi: 10.2196/103353 (PMC13652384; doi:10.2196/103353)
Supplement: Supplementary file 2 [file jmir-v28-e103353-s002.docx]

**Multimedia Appendix 2. Detailed study characteristics**

| Serial Number | Study and location | Study dsign | Study population | Intervention components and setting | Intervention arrangements | Target outcomes and results |
| --- | --- | --- | --- | --- | --- | --- |
| 1 | Smith et al.，2017 （USA) | RCT with two arms. | N=80 IG=42; Mean age：7.6±0.9 CG=38; Mean age：7.2±1.2 Gender: IG:M=30;F=12; CG:M=23;F=15 ADHD symptom：ADHD | Computerized Cognitive Remediation Training (CCRT), physical exercise, and Good Behavior Game (GBG) IG:Integrated Brain(CCRT), Body, and Social (IBBS) Intervention CG:Treatment-As-Usual, TAU Setting：School-based（USA）/nSchool-based | CCRT component:30 min  Body component:45 min Social component:15-30 min USA:4 times a week for 15 weeks;2 hours a week China:3 times a week for 15 weeks，1.5 hours a week follow up:nil | Core Symptoms: (1)Clinician SNAP(Swanson, Nolan, and Pelham Rating Scale):ΔIG=-3（NS,IG VS CG） (2)Parent SNAP:ΔIG=-5.7（NS,IG VS CG） (3)Teacher SNAP:ΔIG=1.7（NS,IG VS CG） (4)Finger Window Forwardsa Forwards:ΔIG=1.1（NS,IG VS CG） (5)Finger Window Backward Forwards:ΔIG=1.1（NS,IG VS CG） cognitive function： CVLT-= California Verbal Learning Test (1)Total learning:ΔIG=7.2（Sig,IG VS CG） (2)Short delay recall:ΔIG=1.8（NS,IG VS CG） (3)Long delay recall:ΔIG=1.6（NS,IG VS CG） Flanker (1)RT=Reaction time:ΔIG=-27（NS,IG VS CG） (2)Error=directional errors:ΔIG=-3.5（NS,IG VS CG） (3）RT variability incongruent:ΔIG=-53（NS,IG VS CG） (4)RTCV=reaction time coefficient of variation:ΔIG=-0.02（NS,IG VS CG） |
| 2 | Bikic et al.，2017 （Denmark） | RCT with two arms. | N=17  (IG=9; CG=8); Age: 14-17 years;Mean age: 15.6±0.99 Gender: M=76.5% ADHD symptom：ADHD com- bined type | Scientific Brain Training (SBT) versus Tetris, the computer game (as an active placebo) IG:Scientific Brain Training(SBT exercises) CG:Tetris Setting：home-based | 30min/day;  5days/week ; for 7 weeks. follow up:Nil | Core Symptoms ADHD-RS (1)Parents：ΔIG=-3.7（NS,IG VS CG） (2)Adolescents：ΔIG=-2.2（NS,IG VS CG） (3)Teachers：ΔIG=1.4（NS,IG VS CG） |
| 3 | Wexler et al.，2020 (USA) | RCT with two arms. | N=73 Mean age：7.4±1.2  IG=55 Mean age：7.3±1.2 CG=18 Mean age：7.5±1.2 Gender: M=48;F=25  IG:M=34;F=21 CG:M=14;F=4 ADHD symptom：ADHD | Computer presentation combined with body cognition training intervention IG:CT exercise(cognitive training） Computer-presented cognitive-training exercises&Physical exercises&Social component GBG CG:treat-ment-as-usual (TAU) Setting：School-based | 45min/time; 3-4times/week; for 15weeks follow up: Nil | Cognitive function (executive function, working memory) Working memory test:ΔIG=4.7（NS,IG VS CG） Flanker reaction time:ΔIG=-0.5（Sig,IG VS CG） Core Symptoms SNAP-Parent:ΔIG=-4.3（Sig,IG VS CG） SNAP-Teacher:ΔIG=0.2（NS,IG VS CG） |
| 4 | Klingberg et al.，2002 （Sweden） | RCT with two arms. | N=14 IG=7 M=6;F=1 Mean age：11±2  CG=7 M=5;F=2 Mean age：11.4±3 ADHD symptom：ADHD | Work memory training IG:Five cognitive tasks； Computerized Training Program CG:placebo/low-dose training Setting：computer-based indoor | least 20 min/day;  4-6 days a week; for at least 5 weeks follow up:Nil | cognitive function Trained visuo-spatial WM（Executive function working memory）：ΔIG=1.72（Sig,IG VS CG） Span board（Executive function working memory）：ΔIG=1.96（Sig,IG VS CG） Stroop task: (1)Accuracy (max 60)：ΔIG=4（Sig,IG VS CG） (2)Time for completion (s)：ΔIG=-10.1（NS,IG VS CG） (3)Raven's progressive matrices：ΔIG=-6.9（Sig,IG VS CG） Choice reaction time task： (1)RT Latency (ms)：ΔIG=14（NS,IG VS CG） (2)Two ± One choice (ms)：ΔIG=-75（NS,IG VS CG） (3)RT standard deviation：ΔIG=-14（NS,IG VS CG） |
| 5 | Klingberg et al.，2005 （Sweden） | RCT with two arms. | N=44 Mean age：9.8±1.3 IG=20; Mean age：9.8±1.4 CG=24; Mean age：9.7±1.3 Gender: IG:M=16;F=4; CG:M=20;F=4; ADHD symptom：ADHD combined andADHD inattentive | Computerized training of working memory IG:computer program(RoboMemo): 90 WM trials  (1)visuospatial WM tasks  (2)verbal tasks CG:90 WM trials  Setting：School/home-based | 90 mins/session;   >25 days of trainin; for 5 weeks Follow up: 12 weeks. | Cognitive function (executive function) Span-board (items) (visual WM)：ΔIG=0.82（Sig,IG VS CG）； Digit-span (items) (verbal WM)：ΔIG=0.46（Sig,IG VS CG） Stroop accuracy (max 60) (response inhibition)：ΔIG=2.5（Sig,IG VS CG） Stroop time (s) (response inhibition)：ΔIG=-1.5（Sig,IG VS CG） Raven accuracy (max 36) (reasoning)：ΔIG=2.2（Sig,IG VS CG） Core Symptoms ADHD parent inatt（Symptoms of inattention）.：ΔIG=-5.7（Sig,IG VS CG） ADHD Parent H/I：ΔIG=-3.2（Sig,IG VS CG） ADHD teacher inatt.：ΔIG=-2（NS,IG VS CG） ADHD teacher H/I：ΔIG=-2.1（NS,IG VS CG） Head movements（Reasoning cognitive function）：ΔIG=372（NS,IG VS CG） |
| 6 | Beck et al.，2010 （USA） | RCT with two arms. | N=52 7-17 years Mean age：11.75 Gender: M=36;F=16; ADHD symptom：combined type (29%) or predominately inattentive type(71%) | Work memory training IG:WM Training Intervention Measures;(Work memory training） Structured clinical interview： Measures of inattention：  Measures of WM.; CG:No intervention;After the posttreat-ment data collection, the waitlist control group receivedthe identical intervention. Setting :Home-based | 25session； 30 - 40 min/session； 6 weeks； Follow up: 16 weeks. | Core Symptoms Conners’ Parent Scales (1)ADHD Index：ΔIG=-8.92（Sig,IG VS CG）CG（Sig,IG VS CG） (2)Cognitive Problems/Inattention ：ΔIG=-8.07（Sig,IG VS CG）CG（Sig,IG VS CG） (3)Hyperactivity：ΔIG=-8.78（Sig,IG VS CG）CG（NS,IG VS CG） (4)Oppositional：ΔIG=-6.04（NS,IG VS CG）CG（NS,IG VS CG） Conners’ Teacher Scales (1)ADHD Index：ΔIG=-4.4（NS,IG VS CG）CG（NS,IG VS CG） (2)Cognitive Problems/Inattention：ΔIG=-3.39（NS,IG VS CG）CG（NS,IG VS CG） (3)Hyperactivity：ΔIG=-3.28（NS,IG VS CG）CG（NS,IG VS CG） (4)Oppositional ：ΔIG=-4.17（NS,IG VS CG）CG（NS,IG VS CG） DSM–IV–TR Inattentive Scale：ΔIG=-2.5（Sig,IG VS CG）CG（Sig,IG VS CG） Cognitive function (executive function) BRIEF Parent Scales (1)Metacognition Index：ΔIG=-8.77（Sig,IG VS CG）CG（NS,IG VS CG） (2)Working Memory：ΔIG=-10.63（Sig,IG VS CG）CG（NS,IG VS CG） (3)Initiate：ΔIG=-9.74（Sig,IG VS CG）CG（SIG,IG VS CG） (4)Monitor：ΔIG=-5.26（NS,IG VS CG）CG（NS,IG VS CG） (5)Organization of Materials：ΔIG=-3.85（NS,IG VS CG）CG（NS,IG VS CG） (6)Plan/Organize ：ΔIG=-8.33（Sig,IG VS CG）CG（Sig,IG VS CG） BRIEF Teacher Scales (1)Metacognition Index：ΔIG=-3.11（NS,IG VS CG）CG（NS,IG VS CG） (2)Working Memory：ΔIG=-5.22（NS,IG VS CG）CG（NS,IG VS CG） (3)Initiate：ΔIG=-4.34（Sig,IG VS CG）CG（NS,IG VS CG） (4)Monitor：ΔIG=-2.63（NS,IG VS CG）CG（NS,IG VS CG） (5)Organization of Materials：ΔIG=-1.74（NS,IG VS CG）CG（NS,IG VS CG） (6)Plan/Organize ：ΔIG=-0.85（NS,IG VS CG）CG（NS,IG VS CG） |
| 7 | Steiner et al.，2011 （USA） | RCT with three arms. | N=41 Mean age：12.4±0.9 NF=13;SCF=13;WLC=15 Gender: M=52.2%; ADHD symptom：ADHD | Computerized attention training IG:Neurofeedback(NF). CG:Waitlist control； Setting :School-based. | 45-minute sessions; twice a week; During the 4-month； Follow up: Nil | Core Symptoms  CRS-R Cognitive Problems/Inattention Scale (1)Parent #1:ΔIG=-8.6（Sig,IG VS CG） (2)Parent #2:ΔIG=-8.2（Sig,IG VS CG） (3)Teacher:ΔIG=-0.1（NS,IG VS CG） (4)Student：ΔIG=-3.7（NS,IG VS CG） CRS-R Hyperactivity Scale (1)Parent #1：ΔIG=-19.2（Sig,IG VS CG） (2)Parent #2：ΔIG=-4.2（NS,IG VS CG） (3)Teacher：ΔIG=0.6（NS,IG VS CG） (4)Student：ΔIG=-1.4（NS,IG VS CG） CRS-R ADHD Index (1)Parent #1：ΔIG=-13.3（Sig,IG VS CG） (2)Parent #2：ΔIG=-5.3（Sig,IG VS CG） (3)Teacher：ΔIG=-1.3（NS,IG VS CG） (4)Student：ΔIG=-2.1（NS,IG VS CG） BASC Hyperactivity Scale (1)Parent #1：ΔIG=-10.3（Sig,IG VS CG） (2)Parent #2：ΔIG=-7.6（NS,IG VS CG） (3)Student：ΔIG=2.7（NS,IG VS CG） BASC Attention Problems Scale (1)Parent #1：ΔIG=-4（Sig,IG VS CG） (2)Parent #2:ΔIG=-2（NS,IG VS CG） (3)Student:ΔIG=-1.5（NS,IG VS CG） Cognitive function (executive function) BRIEF Global Executive Composite Score (1)Parent #1:ΔIG=-4.9（NS,IG VS CG） (2)Parent #2:ΔIG=-6（NS,IG VS CG） (3)Teacher：ΔIG=-7.1（NS,IG VS CG） |
|  | Steiner et al.，2011 （USA） | RCT with two arms. | N=41 Mean age：12.4±0.9 NF=13;SCF=13;WLC=15 Gender: M=52.2%; ADHD symptom：ADHD | Computerized attention training IG:SCF treatment (visual and auditory exercises ) CG:Waitlist control； Setting :School-based. | 45-minute sessions; twice a week; During the 4-month； Follow up: Nil | Core Symptoms  CRS-R Cognitive Problems/Inattention Scale (1)Parent #1:ΔIG=-7.1（Sig,IG VS CG） (2)Parent #2:ΔIG=-5.9（NS,IG VS CG） (3)Teacher:ΔIG=-0.5（NS,IG VS CG） (4)Student：ΔIG=0.2（NS,IG VS CG） CRS-R Hyperactivity Scale (1)Parent #1：ΔIG=-0.6（NS,IG VS CG） (2)Parent #2：ΔIG=4.5（NS,IG VS CG） (3)Teacher：ΔIG=4.8（NS,IG VS CG） (4)Student：ΔIG=0.6（NS,IG VS CG） CRS-R ADHD Index (1)Parent #1：ΔIG=-7.4（Sig,IG VS CG） (2)Parent #2：ΔIG=-2.2（NS,IG VS CG） (3)Teacher：ΔIG=0.8（NS,IG VS CG） (4)Student：ΔIG=-0.7（NS,IG VS CG） BASC Hyperactivity Scale (1)Parent #1：ΔIG=-4.2（NS,IG VS CG） (2)Parent #2：ΔIG=-4.3（NS,IG VS CG） (3)Student：ΔIG=-3.1（NS,IG VS CG） BASC Attention Problems Scale (1)Parent #1：ΔIG=-4.7（Sig,IG VS CG） (2)Parent #2:ΔIG=-1.2（NS,IG VS CG） (3)Student:ΔIG=-6.6（Sig,IG VS CG） Cognitive function (executive function) BRIEF Global Executive Composite Score (1)Parent #1:ΔIG=-8（Sig,IG VS CG） (2)Parent #2:ΔIG=-8.5（NS,IG VS CG） (3)Teacher：ΔIG=-1.2（NS,IG VS CG） |
| 8 | Tucha et al.，2011 （Netherlands） | RCT with three arms. | N=32(ADHD)+16（heathy） IG1=16; Mean age：10.8 ± 0.4 IG2=16; Mean age：11.0 ± 0.6 CG=16; Mean age：10.7 ± 0.4 Gender: IG1:M=11;F=5 IG2:M=11;F=5 CG:M=11;F=5 ADHD symptom：ADHD | Attention Function Training IG1:Attention training： IG2:Visual perception training:  CG: not intervenion Setting:laboratory-based | 8 training sessions ； 1h/training session； twice a week, for 4 weeks  Follow up: nil | Cognitive function  Selective attention (1)Reaction time (ms):ΔIG=-129.6（NS,IG VS CG） (2)Number of omission errors:ΔIG=-1.5（NS,IG VS CG） (3)Number of commission errors:ΔIG=-0.56（NS,IG VS CG） Divided attention (1)Reaction time (ms)：ΔIG=13.2（NS,IG VS CG） (2)Number of omission errors：ΔIG=0.12（NS,IG VS CG） (3)Number of commission errors：ΔIG=-2.13（Sig,IG VS CG） Flexibility (1)Reaction time (ms)：ΔIG=-56.4（NS,IG VS CG） (2)Number of commission errors：ΔIG=-2.75（NS,IG VS CG） |
| 9 | Tamm et al.，2013 （USA） | RCT with two arms. | N=105 IG=54; Mean age：9.1±1.2 CG=51; Mean age：9.5±1.5 Gender: IG:M=36;F=18 CG:M=35;F=16 ADHD symptom：ADHD | Repeated practice of cognitive operations related to attention IG:visual and auditory stimuli（Pay Attention） CG: not intervenion  setting :School based/outpatient clinic | 16 sessions  30min/ session twice a week, for 8 weeks  Follow up: Nil | Core Symptoms  SNAP-IV (1)Inattention-P:ΔIG=-0.71（Sig,IG VS CG） (2)Hyperactivity/Impulsivity-P:ΔIG=-0.37（Sig,IG VS CG） (3)Inattention-C:ΔIG=-0.53（Sig,IG VS CG） (4)Hyperactivity/Impulsivity-C:ΔIG=-0.33（Sig,IG VS CG） (5)Inattention-T:ΔIG=-0.12（NS,IG VS CG） (6)Hyperactivity/Impulsivity-T:ΔIG=-0.27（NS,IG VS CG） BASC-II (1)Externalizing-P:ΔIG=-2.69（NS,IG VS CG） (2)Behavioral Symptoms Index-P:ΔIG=-3.59（NS,IG VS CG） (3)Hyperactivity-P:ΔIG=-5.37（NS,IG VS CG） (4)Attention Problems-P:ΔIG=-4.58（Sig,IG VS CG） (5)Externalizing-T:ΔIG=-2.67（NS,IG VS CG） (6)Behavioral Symptoms Index-T：ΔIG=-4.3（NS,IG VS CG） (7)Hyperactivity-T：ΔIG=-2.96（NS,IG VS CG） (8)Attention Problems-T：ΔIG=-2.15（NS,IG VS CG） Cognitive function (executive function) BRIEF-parent (1)Inhibit：ΔIG=-4.03（NS,IG VS CG） (2)Shift：ΔIG=-4.84（Sig,IG VS CG） (3)Emotion Regulation：ΔIG=-3.09（NS,IG VS CG） (4)Initiate：ΔIG=-7.89（Sig,IG VS CG） (5)Working Memory：ΔIG=-8.85（Sig,IG VS CG） (6)Planning：ΔIG=-8.16（Sig,IG VS CG） (7)Organization：ΔIG=-4.37（Sig,IG VS CG） (8)Monitor：ΔIG=-5.85（Sig,IG VS CG） (9)Behavioral Regulation Index:ΔIG=-4.43（Sig,IG VS CG） (10)Metacognitive Index:ΔIG=-8.44（Sig,IG VS CG） (11)General Executive Composite:ΔIG=-7.34（Sig,IG VS CG） BRIEF-teacher (1)Inhibit:ΔIG=0.95（NS,IG VS CG） (2)Shift:ΔIG=-1.83（NS,IG VS CG） (3)Emotion Regulation：ΔIG=1.31（NS,IG VS CG） (4)Initiate：ΔIG=-0.75（NS,IG VS CG） (5)Working Memory：ΔIG=-2.87（NS,IG VS CG） (6)Planning:ΔIG=-0.74（NS,IG VS CG） (7)Organization:ΔIG=4.04（NS,IG VS CG） (8)Monitor:ΔIG=-0.65（NS,IG VS CG） (9)Behavioral Regulation Index:ΔIG=0.31（NS,IG VS CG） (10)Metacognitive Index:ΔIG=-0.56（NS,IG VS CG） (11)General Executive Composite:ΔIG=-0.23（NS,IG VS CG） DKEFS Color Word Interference (1)Inhibition Scaled Scor:ΔIG=1.68（NS,IG VS CG） (2)Inhibition/Switching Scaled Score:ΔIG=0.88（NS,IG VS CG） (3)Inhibition Errors:ΔIG=1.14（NS,IG VS CG） (4)Inhibition/Switching Errors:ΔIG=2.12（NS,IG VS CG） |
| 10 | Chacko et al.，2014 (USA) | RCT with two arms. | N=85 IG=44; Mean age：8.4±1.4 CG=41; Mean age：8.4±1.3 Gender: IG:M=36;F=8 CG:M=35;F=6 ADHD symptom：ADHD | Cogmed Work memory training（CWMT） IG:CWMT Active：computerized training program  verbal and nonverbal working memory CG：CWMT placebo：a low-level (placebo)  working memory training program Setting:home-based | 30-45 min /session total 25 session ; for 5 weeks Follow up: 3 weeks | Core Symptoms  DBD-IN: Disruptive Behavior Disorder Rating Scale Sum of Inattention Symptoms Parent DBD-IN:ΔIG=-3.15（NS,IG VS CG） Disruptive Behavior Disorder Rating Scale Sum of Hyperactive/Impulsive Symptoms Parent DBD-HI:ΔIG=-2.55（NS,IG VS CG） Inattention Symptoms Teacher DBD-IN:ΔIG=-0.64（NS,IG VS CG） Hyperactive/Impulsive Symptom Teacher DBD-HIs:ΔIG=-1.46（NS,IG VS CG） Continuous Performance Test (1)CPT- Omissions:ΔIG=-1.93（NS,IG VS CG） (2)CPT-Commissions:ΔIG=-8.67（NS,IG VS CG） Cognitive function (executive function) Working memory (1)AWMA; Automatic Working Memory Assessment:ΔIG=25.06（Sig,IG VS CG） (2)AWMA- Spatial Recall:ΔIG=6.48（Sig,IG VS CG） (3)AWMA- Digit Recall:ΔIG=7.99（Sig,IG VS CG） (4)AWMA- Listening Recall:ΔIG=5.35（NS,IG VS CG） Academic achievement（Cognitive function ） (1)WRAT- Word Reading:ΔIG=7.25（NS,IG VS CG） (2)WRAT-Sentence Completion:ΔIG=8.93（NS,IG VS CG） (3)WRAT- Math Computation:ΔIG=8.32（NS,IG VS CG） (4)WRAT- Spelling:ΔIG=10.52（NS,IG VS CG） |
| 11 | Azami et al.，2016 (Iran) | RCT with three arms. | N=34boy Age：7-12years IG1（CACR）=12; IG2（PCACR）=11; IG3（MED ）=11; ADHD symptom：ADHD | Computer-Assisted Cognitive Rehabilitation (CACR) Therapy IG1: computer-assisted cognitive rehabilitation (CACR)  IG2：placebo CACR (PCACR） IG3：sychostimulants (MED) methylphenidate Setting:School-based | IG1 and IG2   90 min /training total 20 training ; for 8 weeks IG310mg/per; 2-3/day;for 8 weeks Follow up: 12 weeks | Core Symptoms  CPT: total correct（持续注意）：ΔIG=6.28（Sig,IG VS CG） SNAP-IV (1)ADHD-PI：ΔIG=-5.90（NS,IG VS CG） (2)ADHD-PHI（hyperactivity–impulsivity symptoms）：ΔIG=-8.198（Sig,IG VS CG） (3)ADHD-C（AD/HD combined symptoms）：ΔIG=-14.09（Sig,IG VS CG） Cognitive function  TOL incorrect responses:executive planning：ΔIG=-10.724（NS,IG VS CG） Span board:visuo-spatial STM（Short-term working memory）：ΔIG=1.364（Sig,IG VS CG） Raven’s Progressive matrices:complex nonverbal reasoning：ΔIG=6.91（Sig,IG VS CG） Backward digit span:verbal STM：ΔIG=1.181（Sig,IG VS CG） |
| 12 | Jones et al.，2018 (USA) | RCT with two arms. | N=80 Age:7-14years Mean age:10.14±2.02 IG=41; Mean age:10.22±2.04 CG=39; Mean age:10.05±2.03 Gender: M=55;F=25 ADHD symptom：ADHD | n-back cognitive training IG:computerized cognitive training task(N-back training) CG:Control training: general knowledge and vocabulary task  Setting：Home-based（16）/social-based（4） | 15min /sessions ; total 20 sessions (16sessions at home ;4times at supervised for about 5 weeks; Follow up: 12 weeks | Core Symptoms assess sustained attention and inhibitory control:CPT：ΔIG=-18.83（Sig,IG VS CG） CPRS–R:L:Inattention and hyperactivity：ΔIG=（Sig,IG VS CG） Cognitive function (executive function) digit span:Working memory：ΔIG=0.06（NS,IG VS CG） BRIEF：Executive functioning：ΔIG=-65.48（NS,IG VS CG） Wood-cock Johnson III Passage Comprehension, Math Applied Problem-Solving,  and Reading Fluency subtests：School-related tasks：ΔIG=0.07（Sig,IG VS CG） |
| 13 | Lan et al.，2018 （China） | RCT with three arms. | N=81 IG1（GEFT）=27; Mean age：10.87±1.32 IG2（SST）=26; Mean age：10.53±0.98 CG（WL）=28 Mean age：10.21±1.08 Gender: I1:M=18;F=9 IG2:M=16;F=10 CG:M=18;F=10 ADHD symptom: ADHD com- bined type | Group executive function training IG1:Group executive function training (GEFT) Computerized EF training&EF elements and EF Meta-cognition IG2:social skills training (SST) social behaviors & social cognition & emotion regulation Setting:School-based. | 60 minutes /session 1 session/weeks 12 weekly sessions Follow up: 12 weeks | Core Symptoms  ADHD Rating Scale (1)Inattention:ΔIG=-5.79（Sig,IG VS CG） (2)Hyperactivity/impulsivity :ΔIG=-8.21（Sig,IG VS CG） CPT-II（Conners’ Continuous performance tasks-II） (1)Commissions :ΔIG=-8.53（Sig,IG VS CG） (2)Omissions :ΔIG=-10.03（Sig,IG VS CG） Cognitive function Primary variable Peer relationship SAICA (1)Interaction with peers:ΔIG=10.43（Sig,IG VS CG） (2)Problems with peers:ΔIG=-7.55（Sig,IG VS CG） Working Memory:ΔIG=0.81（Sig,IG VS CG） WCST（Wisconsin Card Sorting Test） (1）Perseverative Errors:ΔIG=-4.1（NS,IG VS CG） (2)Categories Completed:ΔIG=1.34（NS,IG VS CG） (3)Nonperseverative Errors:ΔIG=-2.32（NS,IG VS CG） Social Skills SSRS (1)Cooperation skills:ΔIG=6.51（Sig,IG VS CG） (2)Responsibility:ΔIG=2.88（Sig,IG VS CG） (3)Assertion:ΔIG=0.65（NS,IG VS CG） (4)Empathy:ΔIG=7.08（Sig,IG VS CG） (5)Self-control:ΔIG=-3.21（Sig,IG VS CG） |
|  | Lan et al.，2018 （China）（a） | RCT with three arms. | N=81 IG1（GEFT）=27; Mean age：10.87±1.32 IG2（SST）=26; Mean age：10.53±0.98 CG（WL）=28 Mean age：10.21±1.08 Gender: IG1:M=18;F=9 IG2:M=16;F=10 CG:M=18;F=10 ADHD symptom: ADHD com- bined type | social skill training IG1:Group executive function training (GEFT) Computerized EF training&EF elements and EF Meta-cognition IG2:social skills training (SST) social behaviors & social cognition & emotion regulation Setting:School-based. | 60 minutes /session 1 session/weeks 12 weekly sessions Follow up: 12 weeks | Core Symptoms  ADHD Rating Scale (1)Inattention:ΔIG=-5.79（Sig,IG VS CG） (2)Hyperactivity/impulsivity :ΔIG=-8.21（Sig,IG VS CG） CPT-II（Conners’ Continuous performance tasks-II） (1)Commissions :ΔIG=-8.53（Sig,IG VS CG） (2)Omissions :ΔIG=-10.03（Sig,IG VS CG） Cognitive function Primary variable Peer relationship SAICA (1)Interaction with peers:ΔIG=10.43（Sig,IG VS CG） (2)Problems with peers:ΔIG=-7.55（Sig,IG VS CG） Working Memory:ΔIG=0.81（Sig,IG VS CG） WCST（Wisconsin Card Sorting Test） (1）Perseverative Errors:ΔIG=-4.1（NS,IG VS CG） (2)Categories Completed:ΔIG=1.34（NS,IG VS CG） (3)Nonperseverative Errors:ΔIG=-2.32（NS,IG VS CG） Social Skills SSRS (1)Cooperation skills:ΔIG=6.51（Sig,IG VS CG） (2)Responsibility:ΔIG=2.88（Sig,IG VS CG） (3)Assertion:ΔIG=0.65（NS,IG VS CG） (4)Empathy:ΔIG=7.08（Sig,IG VS CG） (5)Self-control:ΔIG=-3.21（Sig,IG VS CG） |
| 14 | Estrada-Plana et al.,2019 (Spain.) | RCT with two arms. | N=27 ; IG=13; Mean age：9.46 ± 1.20  CG=14; Mean age：9.50 ± 1.09 Gender: IG:M=7;F=6; CG:M=10;F=4; ADHD symptom:ADHD combined type | Board games focusing on cognitive functions IG:five board games : Alles Tomate Alles Kanone  Spooky Stairs  Out of Mine  Chicken Cha Cha Cha  CG:TAU Setting:laboratory-based | 60min/per; 1 week/per; for 5 weeks  follow up:1 month | Core Symptoms  CPRS-48 (M/SD)： (1)Impulsive-hyperactive：ΔIG=-0.72（NS,IG VS CG） (2)Hyperactivity Index：ΔIG=-2.5（NS,IG VS CG） Cognitive function TMT-A:Shifting：ΔIG=1.3（NS,IG VS CG） TMT-B:Shifting：ΔIG=-1.7（NS,IG VS CG） differenceTMT-B - TMT-A:Shifting：ΔIG=-3.02（NS,IG VS CG） direct digits from WISC-IV:Linguistic STM：ΔIG=1.05（NS,IG VS CG） Corsi block-tapping test:Visuospatial STM：ΔIG=-0.4（NS,IG VS CG） Linguistic keep-track task:Updating linguistic WM：ΔIG=2.04（Sig,IG VS CG） Visuospatial keep track:Updating visuospatial WM：ΔIG=-0.17（NS,IG VS CG） Go-nogo hits:Inhibition：ΔIG=-14.96（NS,IG VS CG） Go-nogo commissions:Inhibition：ΔIG=-0.5（NS,IG VS CG） Go-nogo omissions:Inhibition：ΔIG=14.86（NS,IG VS CG） Go-nogo correct foil:Inhibition：ΔIG=0.5（NS,IG VS CG） Go-nogo TR:Inhibition：ΔIG=78（NS,IG VS CG） CPRS-48 (M/SD): (1)Conduct problems：ΔIG=-0.19（NS,IG VS CG） (2)Learning difﬁculties：ΔIG=-1.4（NS,IG VS CG） (3)Psychosomatic：ΔIG=-0.01（NS,IG VS CG） (4)Anxiety：ΔIG=-0.24（NS,IG VS CG） SDQ (M/SD): (1)Total：ΔIG=-2.02（NS,IG VS CG） (2)Emotional symptoms：ΔIG=-0.54（NS,IG VS CG） (3)Conduct problems：ΔIG=-1.01（Sig,IG VS CG） |
| 15 | Meyer et al.，2020（USA) | RCT with two arms. | N=40 ; IG=20; Mean age：9.84 ± 1.73  CG=20; Mean age：10.82 ± 0.93 Gender: IG:M=13;F=7; CG:M=15;F=5 ADHD symptom:ADHD combined type | Computerized inhibition control (IC) training Computerized training.SST Intervention:Baseball\Fish\Catapult  IG:aptive training CG:non-adaptive training Setting:home-based | 13-15min/day； 5days/week； for 4weeks.  follow up:Nil | Core Symptoms ： (1)Teacher Conners Inattention:ΔIG=-2（NS,IG VS CG） (2)Teacher Conners Hyperactivity:ΔIG=1.2（NS,IG VS CG） (3)Parent Conners Inattention:ΔIG=-3.8（NS,IG VS CG） (4)Parent Conners Hyperactivity:ΔIG=0.8（NS,IG VS CG） (5)Parent SNAPInattention:ΔIG=-1.4（NS,IG VS CG） (6)Parent SNAPHyperactivity:ΔIG=-0.9（NS,IG VS CG） |
| 16 | Medina et al.,2021 (Spain) | RCT with two arms. | N=29 ; IG=15; Mean age：9.2 ± 1.21  CG=14; Mean age：9.71 ± 1.33 Gender: IG:M=13;F=2; CG:M=12;F=2 ADHD symptom:ADHD combined type | The mobile device cognitive stimulation program of artificial intelligence (AI) (KAD_SCL_01) IG:14 games which compose the KAD_SCL_01 CG:3 videogames which were not specifically designed to improve cognitive performance Setting:social-based | 15-20min/per; 3times/week; for 12weeks.  follow up:Nil | Core Symptoms  EDAH scales: (1)Hyperactivity:ΔIG=-3.8（Sig,IG VS CG） (2)Inattention:ΔIG=-4.07（Sig,IG VS CG） (3)Behavioural Disorders:ΔIG=-9.4（Sig,IG VS CG） Cognitive Flexibility: (1)Correct answers:ΔIG=2.6（NS,IG VS CG） (2)Inhibition errors:ΔIG=-1.93（NS,IG VS CG） Cognitive function： BRIEF: (1)Inhibition:ΔIG=-0.8（NS,IG VS CG） (2)Flexibility:ΔIG=-1.2（Sig,IG VS CG） (3)Working Memory:ΔIG=-1.27（Sig,IG VS CG） Cognitive Flexibility: (1)Commissions:ΔIG=-2.4（NS,IG VS CG） (2)Omissions:ΔIG=-0.67（NS,IG VS CG） Inhibition Test: (1)Errors:ΔIG=-1.13（NS,IG VS CG） (2)Self-corrected errors:ΔIG=-1.06（NS,IG VS CG） (3)Response time:ΔIG=64.48（NS,IG VS CG） |
| 17 | Prins et al ，2011，  (Netherlands) | RCT with two arms | N=51 IG=27; Mean age：9.59±1.12 CG=24; Mean age：9.33±1.05 Gender: IG:M=21;F=6; CG:M=21;F=3 ADHD symptom：ADHD | Gamification and computerization of working memory (WM) training IG：Computerized working memory training integrated with game elements CG：Standardized computerized working memory training without game elements Setting:Social based | Enrollment assessment (1 session, duration 50 minutes) Training intervention (3 sessions, once a week, 35-50 minutes per session) Follow up：Nil | Executive function (working memory)： CBTT: visuospatialWM： ΔIG=0.45（Sig,IG VS CG） |
| 18 | Van et al ，2014， (Belgium) | RCT with two arms | N=40 IG=18; Mean age：10.0±0.97 CG=22; Mean age：9.55±1.43 Gender: IG:M=16;F=2; CG:M=17;F=5 ADHD symptom：ADHD | Computerized execution function (EF) correction training of game elements IG：Computerized executive function remediation training named "Braingame Brian" CG：Waiting list design Setting:Social based | includes a total of 25 training sessions, each lasting about 40 minutes, over a period of 5-6 weeks Follow up：9-weeks | Core Symptoms : DBDRS-P： IA： ΔIG=-4.71（NS,IG VS CG） Core Symptoms : DBDRS-P: H/I： ΔIG=-4.3（NS,IG VS CG） Core Symptoms : DBDRS-P: ODD(oppositional deviant disorder)： ΔIG=-1.59（Sig,IG VS CG） Core Symptoms ： DBDRS-P： CD(conduct disorder)： ΔIG=0（NS,IG VS CG） Core Symptoms ： DBDRS-T： IA： ΔIG=-4.87（NS,IG VS CG） Core Symptoms ： DBDRS-T： H/I： ΔIG=-3.2（NS,IG VS CG） Core Symptoms ： DBDRS-T： ODD(oppositional deviant disorder)： ΔIG=-1.07（NS,IG VS CG） Core Symptoms ： DBDRS-T： CD(conduct disorder)： ΔIG=-0.9（NS,IG VS CG） Executive function (inhibitory control)： BRIEF： Inhibition： ΔIG=-1.83（NS,IG VS CG） Executive function (cognitive flexibility)： BRIEF： cognitive flexibility： ΔIG=-0.56（NS,IG VS CG） Executive function (working memory)： BRIEF： working memory： ΔIG=-2.16（NS,IG VS CG） Executive function (metacognition)： BRIEF： metacognition： ΔIG=-9（NS,IG VS CG） Cognitive function (total score of comprehensive cognitive function)： BRIEF： Total： ΔIG=-13.17（NS,IG VS CG） |
| 19 | Dovis et al（A）， 2015， (Netherlands) | RCT with three arms | N=89 IG1=31; Mean age：10.6±1.4 IG2=28; Mean age：10.3±1.3 CG=30; Mean age：10.5±1.3 Gender:not provided ADHD symptom：ADHD | Game-based executive function training（Braingame Brian） IG1：Train all target executive functions IG2：Training inhibition and cognitive flexibility, with working memory acting as a placebo CG：All tasks are in placebo mode Setting:Social based | 25 sessions were completed within 5 weeks, with an average of 5 sessions per week，each session lasts about 35-50 minutes Follow up：3-months | Cognitive function： Stop Task： SSRT： ΔIG1=-38.2 ΔIG2=-38.2 （NS,IG1 VS IG2 ） Cognitive function： Stroop Color and Word Test： STROOP (interfence score)： ΔIG1=-15.6 ΔIG2=-21.1 （NS,IG1 VS IG2 ） Executive function (working memory): Corsi Block-Tapping Board: CBTT-forward: ΔIG1=2.1 ΔIG2=0.6 （Sig,IG1 VS IG2 ） Executive function (working memory): Corsi Block-Tapping Board: CBTT-backward: ΔIG1=1.4 ΔIG2=0.3 （Sig,IG1 VS IG2 ） |
|  | Dovis et al（B）， 2015， (Netherlands) | RCT with three arms | N=89 IG1=31; Mean age：10.6±1.4 IG2=28; Mean age：10.3±1.3 CG=30; Mean age：10.5±1.4 Gender:not provided ADHD symptom：ADHD | IG1：Train all target executive functions IG2：Training inhibition and cognitive flexibility, with working memory acting as a placebo CG：All tasks are in placebo mode Setting:Social based | 25 sessions were completed within 5 weeks, with an average of 5 sessions per week，each session lasts about 35-50 minutes Follow up：3-months | Cognitive function： Stop Task： SSRT： ΔIG1=-38.2（Sig,IG1 VS CG ） Cognitive function： Stroop Color and Word Test： STROOP (interfence score)： ΔIG1=-15.6（NS,IG1 VS CG ） Executive function (working memory): Corsi Block-Tapping Board: CBTT-forward: ΔIG1=2.1（Sig,IG1 VS CG ） Executive function (working memory): Corsi Block-Tapping Board: CBTT-backward: ΔIG1=1.4（Sig,IG1 VS CG ） |
|  | Dovis et al（C）， 2015， (Netherlands) | RCT with three arms | N=89 IG1=31; Mean age：10.6±1.4 IG2=28; Mean age：10.3±1.3 CG=30; Mean age：10.5±1.5 Gender:not provided ADHD symptom：ADHD | IG1：Train all target executive functions IG2：Training inhibition and cognitive flexibility, with working memory acting as a placebo CG：All tasks are in placebo mode Setting:Social based | 25 sessions were completed within 5 weeks, with an average of 5 sessions per week，each session lasts about 35-50 minutes Follow up：3-months | Cognitive function： Stop Task： SSRT： ΔIG2=-38.2（Sig,IG2 VS CG ） Cognitive function： Stroop Color and Word Test： STROOP (interfence score)： ΔIG2=-21.1（NS,IG2 VS CG ） Executive function (working memory): Corsi Block-Tapping Board: CBTT-forward: ΔIG2=0.6（NS,IG2 VS CG ） Executive function (working memory): Corsi Block-Tapping Board: CBTT-backward: ΔIG2=0.3（NS,IG2 VS CG ） |
| 20 | Hahn-Markowitz et al， 2016， (Israel) | RCT with two arms | N=107 IG=54; Mean age：8.4±0.9 CG=53; Mean age：8.6±0.8 Gender: IG:M=37;F=17; CG:M=39;F=15; ADHD symptom：ADHD | Cognitive Function (Cog-Fun) Occupational Therapy Intervention IG：Cognitive function (Cog-Fun) occupational therapy intervention CG：Waiting list design Setting:Social based | For a total of 13 weeks, once a week, for one hour each time Follow up：3-month | executive function P-BRIEF GEC： Executive functions: ΔIG=-5.9（NS,IG VS CG） Core symptoms (inattention, hyperactivity/impulsivity): P-CPRS-R GIT: ADHD symptomatology: ΔIG=-5.5（NS,IG VS CG） living quality P-PedsQL: Quality of life: ΔIG=8.5（NS,IG VS CG） Executive function (inhibitory control): T-BRIEF GEC: Executive functions: ΔIG=1.7（NS,IG VS CG） Core symptoms (inattention, hyperactivity/impulsivity): ADHD symptomatology: ΔIG=1.3（NS,IG VS CG） |
| 21 | kermani et al，2016， (Iran) | RCT with two arms | N=60 IG=30; Mean age：9.9,range(8.5-11) CG=30; Mean age：9.8(8.11-11.2) Gender: IG:M=18;F=12; CG:M=17;F=13; ADHD symptom：ADHD | Structured game-based working memory training IG：Structured game working memory training CG：No intervention waiting design Setting:Social based | A total of 12 weeks, with 2 sessions  per week and 60 minutes per session Follow up：6-month | Executive function (working memory): Psychological Assessment and Rating Scales: verbal working memory: ΔIG=7.4（Sig,IG VS CG） Executive function (working memory): Psychological Assessment and Rating Scales: Visuospatial working memory: ΔIG=4.4（Sig,IG VS CG） Core symptoms (inattention, hyperactivity/impulsivity): Psychological Assessment and Rating Scales: attention deficit and hyperactivity: ΔIG=-28.2（Sig,IG VS CG） Core symptoms (inattention, hyperactivity/impulsivity): Psychological Assessment and Rating Scales: attention deficit: ΔIG=-37.9（Sig,IG VS CG） Cognitive functions (such as social skills, problem behaviors, and academic abilities, etc.): Scores of Dictation and Mathematics: scores of dictation: ΔIG=2（Sig,IG VS CG） Cognitive functions (such as social skills, problem behaviors, and academic abilities, etc.): Scores of Dictation and Mathematics: scores of mathematic: ΔIG=3.2（Sig,IG VS CG） |
| 22 | Bul et al，2016， (Netherlands) | RCT with two arms | N=170 IG=88; Mean age：9.85±1.26 CG=82; Mean age：9.89±1.28 Gender: IG:M=70;F=18; CG:M=67;F=15 ADHD symptom: | The online serious game called "Plan-It Commander" IG:Conventional treatment + Serious game intervention(0-10 weeks)  Conventional treatment(10-20 weeks) CG:Conventional treatment(0-10 weeks)  Conventional treatment + Serious game intervention(10-20 weeks) Setting:Social based | A total of 20 weeks, with approximately 3 sessions per week, and each session lasting up to 65 minutes Follow up：10-week | Executive function (working memory): P-BRIEF: Plan/Organize: ΔIG=-0.83（Sig,IG VS CG） Cognitive functions (such as social skills, problem behaviors, and academic abilities, etc.): P-SSRS: Cooperation: ΔIG=-0.64（NS,IG VS CG） Executive function (working memory): P-BRIEF: Working Memory: ΔIG=-0.92（Sig,IG VS CG） Cognitive functions (such as social skills, problem behaviors, and academic abilities, etc.): P-SSRS: Assertiveness: ΔIG=-0.38（NS,IG VS CG） Cognitive functions (such as social skills, problem behaviors, and academic abilities, etc.): P-SSRS: Responsibility: ΔIG=-0.64（Sig,IG VS CG） Cognitive functions (such as social skills, problem behaviors, and academic abilities, etc.): P-SSRS: Self-Control: ΔIG=-0.02（NS,IG VS CG） executive function P-SSRS: Total: ΔIG=-1.66（NS,IG VS CG） Executive function (working memory): T-BRIEF: Plan/Organize: ΔIG=-0.64（NS,IG VS CG） Executive function (working memory): T-BRIEF: Working Memory: ΔIG=-0.82（NS,IG VS CG） executive function T-SSRS: Total: ΔIG=-0.59（NS,IG VS CG） |
| 23 | Bikic et al ，2018， (Denmark) | RCT with two arms | N=70 IG=35; Mean age：9.77±1.97 CG=35; Mean age：10.14±1.52 Gender: IG:M=29;F=6; CG:M=30;F=5 ADHD symptom: | A computer training program for multiple cognitive functions ACTIVATE™ IG:ACTIVATE™ computerized cognitive training + usual care (TAU) CG:Conventional treatment (TAU) Setting:Home based | For a total of 7 weeks, with 5 sessions  per week, each lasting 30 minutes Follow up：12-week and 24-week | Executive function (inhibitory control): P-BRIEF: P-BRIEF impulse inhibition: ΔIG=-4（NS,IG VS CG） Executive function (cognitive flexibility): P-BRIEF: P-BRIEF fexibility: ΔIG=-2.24（NS,IG VS CG） Executive function (inhibitory control): P-BRIEF: P-BRIEF emotional control: ΔIG=-3.12（NS,IG VS CG） executive function P-BRIEF: P-BRIEF AI: ΔIG=-3.86（NS,IG VS CG） Executive function (working memory): P-BRIEF: P-BRIEF initiation: ΔIG=-4.11（NS,IG VS CG） Executive function (working memory): P-BRIEF: P-BRIEF working memory: ΔIG=-5.49（NS,IG VS CG） Executive function (working memory): P-BRIEF: P-BRIEF plan/organize: ΔIG=-2.81（NS,IG VS CG） Executive function (working memory): P-BRIEF: P-BRIEF organize materials: ΔIG=-1.14（NS,IG VS CG） Executive function (inhibitory control): P-BRIEF: P-BRIEF monitor: ΔIG=-2.63（NS,IG VS CG） Executive function (working memory): P-BRIEF: P-BRIEF metacognitive index: ΔIG=-6.35（NS,IG VS CG） executive function P-BRIEF: P-BRIEF total ΔIG=-4.86（NS,IG VS CG） Executive function (inhibitory control): T-BRIEF： T-BRIEF impulse inhibition: ΔIG=4.9（NS,IG VS CG） Executive function (cognitive flexibility): T-BRIEF： T-BRIEF fexibility: ΔIG=6.29（NS,IG VS CG） Executive function (inhibitory control): T-BRIEF： T-BRIEF emotional control: ΔIG=5.37（NS,IG VS CG） Executive function (working memory): T-BRIEF： T-BRIEF initiation: ΔIG=1.63（NS,IG VS CG） Executive function (working memory): T-BRIEF： T-BRIEF working memory: ΔIG=1.28（NS,IG VS CG） Executive function (working memory): T-BRIEF： T-BRIEF plan/organize: ΔIG=2.92（NS,IG VS CG） Executive function (working memory): T-BRIEF： T-BRIEF organize materials: ΔIG=-0.25（NS,IG VS CG） Executive function (inhibitory control): T-BRIEF： T-BRIEF monitor: ΔIG=2.19（NS,IG VS CG） Executive function (working memory): T-BRIEF： T-BRIEF metacognitive index: ΔIG=1（NS,IG VS CG） executive function T-BRIEF： T-BRIEF total: ΔIG=3.83（NS,IG VS CG） Core symptoms (inattention, hyperactivity/impulsivity): P-ADHD-I: ΔIG=-2.95（NS,IG VS CG） Core symptoms (inattention, hyperactivity/impulsivity): P-ADHD-H: ΔIG=-3.13（NS,IG VS CG） Core symptoms (inattention, hyperactivity/impulsivity): P-ADHD-ODD/CD: ΔIG=-0.75（NS,IG VS CG）Core symptoms (inattention, hyperactivity/impulsivity): P-ADHD-total: ΔIG=-7.2（NS,IG VS CG） Core symptoms (inattention, hyperactivity/impulsivity): T-ADHD-I: ΔIG=0.93（NS,IG VS CG） Core symptoms (inattention, hyperactivity/impulsivity): T-ADHD-H: ΔIG=0.96（NS,IG VS CG） Core symptoms (inattention, hyperactivity/impulsivity): T-ADHD-ODD/CD: ΔIG=1.35（NS,IG VS CG） Core symptoms (inattention, hyperactivity/impulsivity): T-ADHD-total: ΔIG=7.01（NS,IG VS CG） Core symptoms (inattention, hyperactivity/impulsivity): P-WFIRS-total: ΔIG=-0.14（NS,IG VS CG） |
| 24 | García-Redondo et al， 2019， (Spain) | RCT with two arms | N=44 IG=24; Mean age：11.71±2.83 CG=20; Mean age：11.71±2.83 Gender: IG:M=14;F=10; CG:M=13;F=7 ADHD symptom:ADHD | Two serious games based on the ToI method（“Boogies Academy”“Cuibrain”） IG:Students aged 6-10 use "Boogies Academy",  while those aged 11-16 use "Cuibrain" CG:Waiting list design Setting:Social based | A total of 28 training sessions, 2  sessions per week, 10 minutes per session, with a total intervention period of 14 weeks Follow up：Nil | Core symptoms (inattention, hyperactivity/impulsivity): D2 Attention Test: Total-quality of attention: ΔIG=82.2（Sig,IG VS CG） Core symptoms (inattention, hyperactivity/impulsivity): D2 Attention Test: Concentration: ΔIG=36.57（Sig,IG VS CG） Core symptoms (inattention, hyperactivity/impulsivity): D2 Attention Test: Correct responses: ΔIG=35.72（Sig,IG VS CG） Core symptoms (inattention, hyperactivity/impulsivity): EDAH scale: Attention deﬁcit: ΔIG=0.17（NS,IG VS CG） Core symptoms (inattention, hyperactivity/impulsivity): EDAH scale: Hyperactivity/impulsivity : ΔIG=-0.48（NS,IG VS CG） Core symptoms (inattention, hyperactivity/impulsivity): EDAH scale: Attention deﬁcit and hyperactivity/impulsivity symptoms: ΔIG=0（NS,IG VS CG） |
| 25 | Rajabi et al，2019， (Iran) | RCT with two arms | N=32 IG=16 Mean age:10.2±1.03 CG=16 Mean age:10.05±0.83 ADHD symptom:ADHD | Neurofeedback and game-based cognitive training  IG:Neurofeedback (NF)and Gamified Cognitive Training (CT) CG:Waiting list design Setting:School based intervention | A total of 30 training sessions, 3 times  per week, 45 minutes per session, with  a total duration of 3 months Follow up：Nil | Core symptoms (inattention, hyperactivity/impulsivity): IVA=Integrated Visual and Auditory Continuous Performance: VA = Visual Attention: ΔIG=28.2（Sig,IG VS CG）Core symptoms (inattention, hyperactivity/impulsivity): IVA=Integrated Visual and Auditory Continuous Performance: AA = Auditory Attention ΔIG=18.6（NS,IG VS CG） Core symptoms (inattention, hyperactivity/impulsivity): IVA=Integrated Visual and Auditory Continuous Performance: TotalA=total Attention: ΔIG=18.6（Sig,IG VS CG） Core symptoms (inattention, hyperactivity/impulsivity): CPRSR=Conners Parent Rating Scales-Revised: Attention Deficit: ΔIG=-2.3（Sig,IG VS CG） Core symptoms (inattention, hyperactivity/impulsivity): CTRS-R=Conners Teacher Rating Scales-Revised: Attention Deficit: ΔIG=-3.9（Sig,IG VS CG） Executive function (visual inhibition control): IVA=Integrated Visual and Auditory Continuous Performance: VRC=Visual response control; ΔIG=26.29（Sig,IG VS CG） Executive function (auditory inhibition control): IVA=Integrated Visual and Auditory Continuous Performance: ARC=Auditory response control: ΔIG=30.5（NS,IG VS CG） Executive function (summarization of inhibition and control): IVA=Integrated Visual and Auditory Continuous Performance: Total RC=total response control: ΔIG=30（Sig,IG VS CG） Core symptoms (inattention, hyperactivity/impulsivity): CPRS-R: H/I: ΔIG=-1.9（Sig,IG VS CG） Core symptoms (inattention, hyperactivity/impulsivity): CTRS-R: H/I: ΔIG=-4.21（Sig,IG VS CG） |
| 26 | Benzing et al.，2018 (Switzerland) | RCT with two arms. | N=46  IG=24 Mean Age:10.46±1.35. Gender: M=83.3% CG=22 Mean Age:10.50±1.41 Gender:M=81.8% ADHD symptoms:combination of Inattantionan and Hyperactivity-Impulsivity | Electronic fitness simulation game IG Intervention: physical activity of moderate intensity:play an exergame CG:sedentary :watching videos setting  Family-based | Testing was scheduled between 1 p.m. and 4 p.m. /15 minutes（including a short break of approximately 1 minute) follow up:Nil | executive function Flanker Task reaction times (1)Congruent:ΔIG=-63ms(NS,IG vs.CG) (2)Inhibition:ΔIG=-139ms(Sig,IG vs.CG) (3)Switching:ΔIG=-122ms(Sig,IG vs.CG) (4)Global switch costs:ΔIG=-100ms(Sig,IG vs.CG) executive function Color span backwards (1)Working memory:ΔIG=0(NS,IG vs.CG) |
| 27 | Benzing et al，2019 （Switzerland） | RCT with two arms. | N=51  IG=28 Mean Age:10.46±1.30 Gender: M=86.4% CG=23 Mean Age:10.39±1.44 Gender:M=81.8% ADHD symptoms:combination of Inattantionan and Hyperactivity-Impulsivity | Interactive motion-sensing game intervention IG Intervention: play an exergame CG:waiting-list  setting  Family-based | 8 weeks (3 times a week for at least 30 minutes)  follow up:Nil | athletic ability Motor ability performance(German Motor Test) (1)Balancing:ΔIG=4.74(NS,IG vs.CG) (2)Jumping:ΔIG=6.8(Sig,IG vs.CG) (3)Sit-ups:ΔIG=0(NS,IG vs.CG) (4)Push-ups:ΔIG=9.65(Sig,IG vs.CG) (5)Long-jump:ΔIG=2.07(NS,IG vs.CG) (6)Stand and reach:ΔIG=-0.34(NS,IG vs.CG) (7)Total:ΔIG=5.34(Sig,IG vs.CG) Core symptom ADHD Symptoms (DSM-IV) (1)Hyperactivity:ΔIG=-1.94(NS,IG vs.CG) (2)Inattentiveness:ΔIG=-2.08(NS,IG vs.CG) (3)Combined:ΔIG=-2.05(NS,IG vs.CG) executive function Simon Task (1)Inhibition:ΔIG=-53ms(Sig,IG vs.CG) executive function Flanker Task (1)Switching:ΔIG=-181ms(Sig,IG vs.CG) executive function Color span backward (1)Updating:ΔIG=1.54(NS,IG vs.CG) |
| 28 | Ji et al.2023 (China) | RCT with two arms. | N=30 IG=16 Mean Age:9.00±1.46 Gender: M=14 CG=14 Mean Age:8.85±1.63 Gender:M=12 ADHD symptoms:combination of Inattantionan and Hyperactivity-Impulsivity | Interactive electronic motion-sensing game IG Intervention: EXG: exergaming group CG:BEG: bicycle exercise group.  setting  Social-based（3 SK Pediatric Mental Health Facilities） | 3days/week, 50 min/day，reserve for 4 weeks follow up:Nil | Cognitive function / Executive function Frankfurter Aufmerksamkeits-Inventar (FAIR)-attention (1)Selective attention:ΔIG=78.62(Sig,T1 vs.T2) (2)Self-control:ΔIG=0.05(Sig,T1 vs.T2) (3)Sustained attention:ΔIG=91.11(Sig,T1 vs.T2) cognitive function Go/No-go Task (1)Go stimulus:ΔIG=-2.9(Sig,IG vs.CG) (2)No-go stimulus:ΔIG=-2.06(Sig,IG vs.CG) |
| 29 | Zhao et al. 2024 (China) | RCT with two arms. | N=80 Mean age：8.4±1.3 IG=40; Mean age：8.5±1.5 CG=40; Mean age：8.3±1.1 Gender: IG:M=34;F=6; CG:M=29;F=11; ADHD symptom：ADHD | Interactive Augmented Reality (AR) exercise therapy IG: BrainFit( a novel digital intervention combining gamified cognitive and exercise training) CG:Waiting Setting:Hospital-based | 30min/session; Total 12 sessions; 3 sessions / week  For 4 weeks follow up:nil | Core symptom SNAP-IV (1)Total:ΔIG=-16.45（Sig,IG VS CG） (2)Inattention:ΔIG=-5.57（Sig,IG VS CG） (3)Hyperactivity/Impulsivity:ΔIG=-6（Sig,IG VS CG） executive function BRIEF (1)Behavior Regulation Index:ΔIG=-5.48（NS,IG VS CG） (2)Metacognition Index:ΔIG=-8.08（Sig,IG VS CG） (3)Global Executive Composite:ΔIG=-7.5（Sig,IG VS CG） |
| 30 | Cho et al.2002 (Korea) | RCT with three arms. | N=26 IG1=8; Mean age：13±0.82 IG2=9; Mean age：15.11±0.6 CG=9; Mean age：14.67±0.5 Gender: ADHD symptom：ADHD | Virtual Reality (VR) Cognitive Training IG1:cognitive training program（Virtual Reality Cognitive Training (VRCT)） IG2:Non-VR Cognitive Training (NVRCT) CG:Waiting Setting：School-based | 20 min/sessions ; eight sessions  over 2 weeks follow up:nil | CPT  Core symptom Number of correct answers:ΔIG=9.37（Sig,IG VS CG） Number of omission errors:ΔIG=-9.38 Number of commission errors:ΔIG=-6.13 cognitive function Reaction time (T score):ΔIG=-0.96 Perceptual sensitivity (d9, T score):ΔIG=-8.05（Sig,IG VS CG） Response bias:ΔIG=-15.25（Sig,IG VS CG） |
| 31 | Cho et al .2004 (Korea) | RCT with three arms. | N=28  age：14-18 years IG1=10; IG2=9; CG=9; Gender:Man ADHD symptom：ADHD | Neurofeedback (EEG biofeedback) combined with virtual reality (VR) technology IG1:neurofeedback&head-mounted display (HMD) and a head tracker IG2:neurofeedback&computer monitor with a fixed viewpoint. CG:Waiting Setting：Lab-based（Medical Center） | about20 min/ession； eight sessions ； over a 2-week  follow up:nil | Core symptom CPT (1)Omissions:ΔIG=-11.33（Sig,IG VS CG） (2)Commissions:ΔIG=-6.77（NS,IG VS CG） |
| 32 | Weerdmeester et al.2016 (Netherlands) | RCT with two arms. | N=73 IG=37; Mean age：9.84±1.71 CG=36; Mean age：9.69±1.79 Gender: IG:M=30;F=7; CG:M=28;F=8 ADHD symptom：ADHD | Whole-body video game intervention IG:Game-Based ADHD Intervention CG:commercial age-appropriate game Setting：School-based | 15 minutes/session;  six sessions ; during 3 weeks; follow up:nil | Core symptom ADHD VragenLijst（AVL) (1)Total:ΔIG=-2.97（Sig,IG VS CG） (2)Hyperactivity:ΔIG=-1.45（Sig,IG VS CG） (3)Impulsivity:ΔIG=-1.18（NS,IG VS CG） (4)Inattention:ΔIG=-0.58（NS,IG VS CG） cognitive function Go/no-go (1)Hits:ΔIG=-5.39（Sig,IG VS CG） (2)False alarms:ΔIG=13.3（Sig,IG VS CG） MABC-2-NL（athletic ability） Fine motor skills:ΔIG=0.73（NS,IG VS CG） Gross motor skills:ΔIG= 0.81（NS,IG VS CG） |
| 33 | KIM et al,2020 ( Korea) | RCT with two arms. | N=40  IG=20  Mean age=8.6  CG=20  Mean age= 8.75  Gender: IG:M=17;F=3  CG:M=18 F=2 ADHD symptom:ADHD Combined type | Eye-contact game based on mixed reality (MR) IG:MR HMD CG:no intervention setting Korea：School-based | 1)IG:MR HMD:30min/per; 15 sessions over 6 weeks  2)follow up:Nil | (1) Core symptom ATA TAST:Omissions:ΔIG=-17.6 （Sig,IG VS CG） Commissions:ΔIG=-11（NS,IG VS CG） |
| 34 | Bioulac et al.2018 ( France) | RCT with three arms | N=51 IG1=16 Mean age=9.5±1.2 IG2=19 Mean age=8.4±0.99 CG=19 Mean age=8.8±1.07 Gender： IG1：F=2 M=14 IG2：F=0 M=16 CG：F=9 M=11 ADHD symptom:ADHD Combined type | Virtual Classroom Cognitive Rehabilitation IG1:VCCR（virtual classroom cognitive remediation） IG2:MED（methylphenidate group) CG：PPT  setting France:Community-based | 1)IG^1^:VCCR:30min/per 2times/week 12sessions over 6weeks IG^2^:MED:8weeks CG:PPT :30min/per  12sessions over 6-8weeks  2)follow up:Nil | Core symptom (1)ADHD-RS Total:ΔIG=-5.06（Sig,IG VS CG） Inattention:ΔIG=-3.25（Sig,IG VS CG） Hyperactivity:ΔIG=1.82（Sig,IG VS CG） (2)Virtual classroom tast Correct hits:ΔIG=18.44（Sig,IG VS CG） CommissionsZ:ΔIG=-11（Sig,IG VS CG） （3）CPT Omissions:ΔIG=2.18（Sig,IG VS CG） Commissions:ΔIG=-3.27（Sig,IG VS CG） cognitive function CommissionsZ:ΔIG=-11（Sig,IG VS CG） |
| 35 | Chang et al.2022 (Taiwan) | RCT with three arms | N=48 IG1=16 Mean age=8.31±1.30 IG2=16 Mean age=8.38±1.20 CG=16 Mean age=8.38±1.31 Gender: IG1:M=13 F=3 IG2:M=13 F=3 CG:F=13 F=3 ADHD symptom: ADHD | IG1:Actual table tennis group Simulated (interactive motion-sensing game) table tennis training IG2:Simulated (Exergame) Table Tennis Training  CG:no intervention setting China：School-based | 1)3*1h/week 32 sessions over 12 weeks  2)follow up:Nil | executive function （1）Graphomotor Function Tests（Handwriting Performance and Movement Tests）： Short Paragraph Copy:ΔIG=11.37（Sig,IG VS CG） Response Time:ΔIG=-41.47（Sig,IG VS CG） Time to Automation:ΔIG=-6.27（Sig,IG VS CG） Mean Stroke Velocity:ΔIG=6.73（Sig,IG VS CG） （2）The Stroop Test（Primary measurement suppression control）： Stroop Color:ΔIG=3.25（NS,IG VS CG） Stroop Color-Word:ΔIG=10.69（Sig,IG VS CG） （3）Wisconsin Card Sorting Test（Mainly tests cognitive functions） Total Errors:ΔIG=-8.12（Sig,IG VS CG） Perseverative Responses:ΔIG=-2,43（NS,IG VS CG） Perseverative Errors:ΔIG=-2.06（NS,IG VS CG） Non-Perseverative Errors:ΔIG=-4.69（Sig,IG VS CG） |
| 36 | Rodrigo-Yanguas et al .2023 (Spain) | RCT with three arms. | N=89 IG1=31 Mean Age:14.3 ±2.24 Gender: M=27;f=4  IG2=24 Mean Age:13.81 ±1.93 Gender: M=19;F=5  CG=34 Mean Age:14.83 ±2.47 Gender:M=18;F=16; ADHD symptoms:ADHD | Virtual reality serious game of chess IG1:Cognitive intervention with TSTM( The Secret Trail of Moon) IG2:Cognitive intervention with TC（chess） CG:phone communications setting:School-based | 2 weekly sessions ； once per week follow up:3 months（had 3 groups assessed weekly,ending at D90.） | Core symptom SNAP (1)Inattentive：ΔIG=-1（NS,IG VS CG） (2)Hyperactive/Inattentive：ΔIG=-0.25（NS,IG VS CG） executive function BRIEF-2 (1)Inhibition：ΔIG=0.75（NS,IG VS CG） (2)Self-supervision：ΔIG=0（NS,IG VS CG） (3)Flexibility：ΔIG=-1.25（NS,IG VS CG） (4)Emotional control：ΔIG=0.5（NS,IG VS CG） (5)Initiative：ΔIG=0（NS,IG VS CG） (6)Working Memory：ΔIG=-1（NS,IG VS CG） (7)Planning / Organization：ΔIG=-0.25（NS,IG VS CG） (8)Task supervision：ΔIG=1.25（NS,IG VS CG） (9)Material organization：ΔIG=-0.25（NS,IG VS CG） cognitive function CPRS-HI：ΔIG=-3（NS,IG VS CG） CPT-3 (1)Response Style：ΔIG=7.75（NS,IG VS CG） (2)Detectability：ΔIG=2.25（NS,IG VS CG） (3)Omissions：ΔIG=0（NS,IG VS CG） (4)Comissions：ΔIG=-3.25（NS,IG VS CG） (5)Perseverations：ΔIG=0（NS,IG VS CG） (6)Reaction time：ΔIG=1.75（NS,IG VS CG） (7)Reaction time standard deviation：ΔIG=-1（NS,IG VS CG） (8)Variability：ΔIG=1.75（NS,IG VS CG） (9)Block change：ΔIG=1（Sig,IG VS CG） (10)Interstimulus change：ΔIG=4.75（Sig,IG VS CG） |
| 37 | Wong et al. 2024 (China) | RCT with three arms. | N=90 IG1=30 Mean Age:8.63 ±1.90 Gender: M=23  IG2=30 Mean Age:8.30 ±1.70 Gender: M=26  CG=30 Mean Age:8.67 ±1.45 Gender:M=24 ADHD symptoms:combination of Inattantionan and Hyperactivity-Impulsivity | Social skills training based on virtual reality IG 1Intervention: virtual reality training IG2traditional social skills training. CG:waitlist control.  setting  School-based | IG Duration： 12 sessions for 3 weeks/ each session lasting 20 minutes. follow up:Nil | cognitive function Social Skills Improvement System-Rating Scales (1)Self-control:ΔIG=1.76(Sig,IG vs.CG) (2)Initiative:ΔIG=0.7(Sig,IG vs.CG) (3)Cooperation:ΔIG=0.1(NS,IG vs.CG) cognitive function Riggio basic social skills assessment (1)Clinical psychological assessment of social skills:ΔIG=11.97(Sig,IG vs.CG) executive function Behavior Rating Inventory of Executive Function-Parents (1)Inhibition:ΔIG=0.8 (2)Emotional control:ΔIG=-1.7(Sig,IG vs.CG) |
|  | Wong et al. 2024 (China) | RCT with three arms. | N=90 IG1=30 Mean Age:8.63 ±1.90 Gender: M=23  IG2=30 Mean Age:8.30 ±1.70 Gender: M=26  CG=30 Mean Age:8.67 ±1.45 Gender:M=24 ADHD symptoms:combination of Inattantionan and Hyperactivity-Impulsivity | IG 1Intervention: virtual reality training IG2traditional social skills training. CG:waitlist control.  setting  School-based | IG Duration： 12 sessions for 3 weeks/ each session lasting 20 minutes. follow up:Nil | cognitive function Social Skills Improvement System-Rating Scales (1)Self-control:ΔIG=1.76(Sig,IG vs.CG) (2)Initiative:ΔIG=0.7(Sig,IG vs.CG) (3)Cooperation:ΔIG=0.1(NS,IG vs.CG) cognitive function Riggio basic social skills assessment (1)Clinical psychological assessment of social skills:ΔIG=11.97(Sig,IG vs.CG) executive function Behavior Rating Inventory of Executive Function-Parents (1)Inhibition:ΔIG=0.8(Sig,IG vs.CG) (2)Emotional control:ΔIG=-1.7(Sig,IG vs.CG) |
| *Note*: RCT means randomized controlled trial; IG means intervention group; CG means control group; F means female; M means male; Sig means significance; NS means non-significance. | | | | | | |
